# Optimized design of battery pole control system based on dual-chip architecture

**DOI:** 10.1371/journal.pone.0264285

**Published:** 2022-05-11

**Authors:** Yanjun Xiao, Shuhan Deng, Weiling Liu, Wei Zhou, Feng Wan

**Affiliations:** 1 School of Mechanical Engineering, Tianjin Key Laboratory of Power Transmission and Safety Technology for NewEnergy Vehicles, Hebei University of Technology, Tianjin, China; 2 Career Leader intelligent control automation company, Suqian, Jiangsu Province, China; Jiangsu University of Science and Technology, CHINA

## Abstract

At present, the global demand for lithium batteries is still in a high growth state, and the traditional lithium battery pole mill control system is still dominated by ARM (Artificial Intelligence Enhanced Computing), DSP (Digital Signal Processing), and other single-chip control methods. There are problems such as poor anti-interference ability and insufficient real-time online analysis of production data. This paper adopts the dual-chip control system architecture based on "ARM+DSP", starting from the mechanical characteristics and operating signal features of the pole mill. The hardware system adopts a three-unit joint control hardware structure, which separates the control unit from the data processing unit and improves the operation of the system. The software system adopts fuzzy PID algorithm to realize deflection control and tension control, and verifies that the Fuzzy PID (Proportion Integration Differentiation) control algorithm can effectively improve the anti-interference ability of the deflection system and tension system. The results show that the data loss rate is low with the SPI communication between DSP and ARM. The tension error of the "ARM+DSP" control system does not exceed 5%, and the deviation of the correction band is within ±4mm. The dedicated dual-chip hardware architecture effectively improves the robustness and operation efficiency of the pole mill, solves the problem of low tension control accuracy, and provides a theoretical basis for the application of the dual-roll mill.

## Introduction

**Fig 1:**
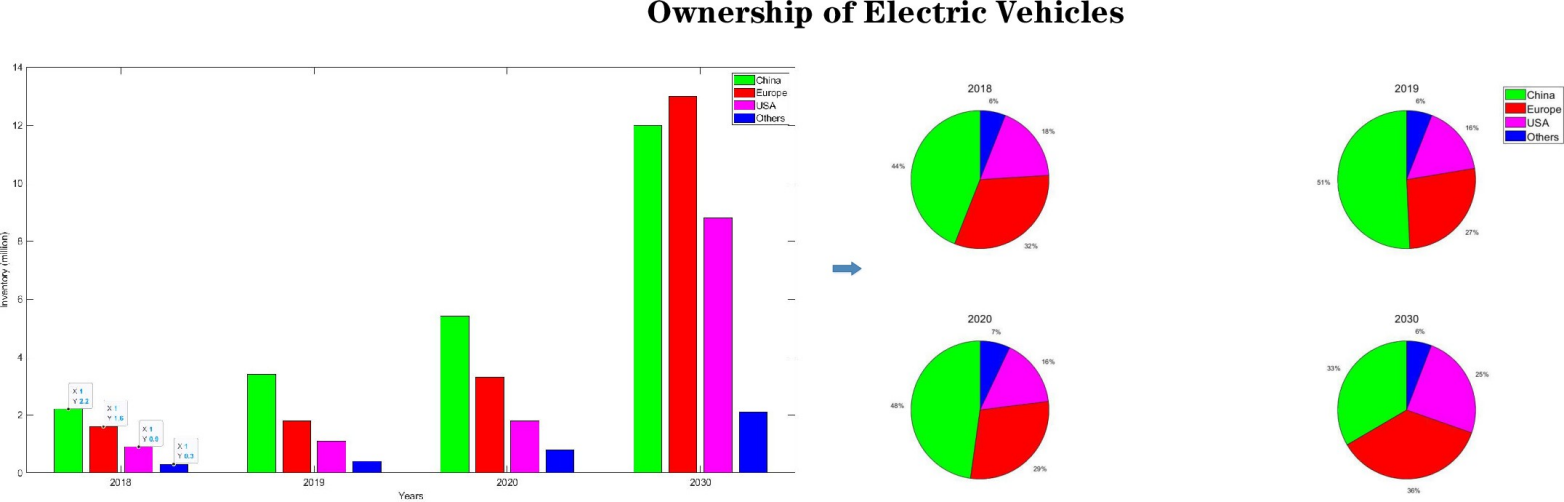
Global EV ownership.

**Fig 2:**
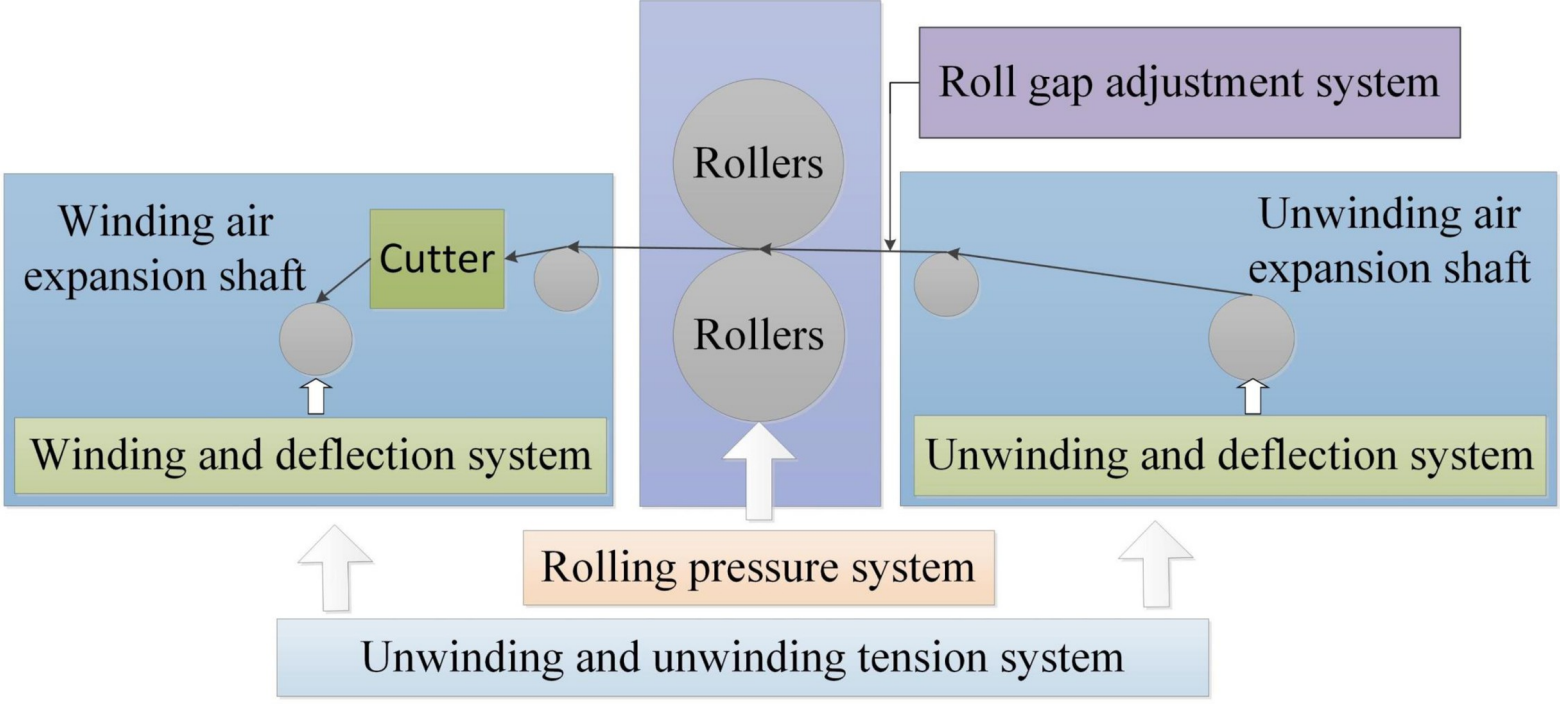
Schematic diagram of lithium battery pole plate rolling mill system.

The level of development of modern manufacturing equipment determines the industrial strength of a country due to the characteristics of high automation, high efficiency, high precision, high reliability, high flexibility, low energy consumption, green and environmental protection [1]. In recent years, lithium batteries have been widely used in cell phones, tablet PCs and electric vehicles with their unique advantages [1,2]. According the report of *Global EV (Electric Vehicles) Outlook 2021*, we considered that the electric vehicle market remains huge, as shown in Fig 1, from the global market, the global electric vehicle ownership is in a high growth stage. In the three years of 2018–2020, the EV ownership in China, Europe and the U.S. grew steadily, and the global EV ownership also reached 13.1million. According to prediction of the IEA (International Energy Agency), the global EV ownership will reach 35million in 2030, among which China and Europe will reach 12 million and 13 million in China and Europe [3]. Therefore, lithium batteries, as the core component of electric vehicles, still have a large demand, and it is necessary to optimize the production and assembly process of lithium batteries [4] The specific workflow of the lithium battery electrode mill is shown in Fig 2, and its main manufacturing process is to release the unrolled electrode by the unwinding system, to coat the lithiated active substance on the carrier surface with aluminum foil or copper foil as the base carrier, and then to compact it by the main roll, and finally to recycle the rolled lithium battery electrode by the winding system [5]. The pole mill equipment is a multi-motor controlled transmission machine. And for the multi-motor control and harsh production environment, multi-coupled interference signals such as high frequency, RF (Radio Frequency), and spikes are generally encountered in its digital hardware system integration design [6]. Traditional machinery and equipment control units use industrial control machines or embedded control, represented by PLC (Programmable Logic Controller), ARM, and DSP. The reliability of PLC makes it ideal for applications in production plants with complex environments, plus it has protection circuits and self-diagnostic functions, making it widely used in industrial control. However, its weak Internet communication cannot meet the needs of smart manufacturing and digital factory. The most important feature of ARM is its high reconfigurability and low power consumption, which can realize high-speed data exchange in industrial sites. The DSP has a high computing speed and can ensure the reliability of data storage. But its control capability is not high, and it cannot control high-power power devices alone.

The pole mill is a multi-motor transmission equipment with more field disturbances. The traditional single-chip control method cannot meet the two requirements of stability and reconfigurability at the same time. Stability is necessary to ensure the normal operation of the mechanical equipment, while reconfigurability enables the pole chip mill to automatically adjust its production state and execute the preset tasks autonomously [7]. Unlike conventional strip rolling, the rolling process has not yet formed a systematic process mathematical model system and automatic control system due to the special laminar structure and particle coating characteristics of the pole sheet, and still relies on manual empirical settings [8]. In order to improve the stability of the control system, most of the research focuses on PLC control system, but it has the disadvantages of insufficient data transmission capability and poor reconfigurability. In order to improve the stability of the control system, most of the research focuses on PLC control system, but it has the disadvantages of insufficient data transmission capability and poor reconfigurability. With the rise of embedded control systems, researchers have started to adopt single-chip control systems with ARM as the core to improve the anti-interference capability of ARM systems and make them suitable for industrial sites. However, since simultaneous system control and data processing will accelerate the damage of the ARM chip, a cascade structure is considered to divide the system functions and adopt the "ARM+DSP" dual-chip control system to improve the system operation speed and lifetime.

**Fig 3:**
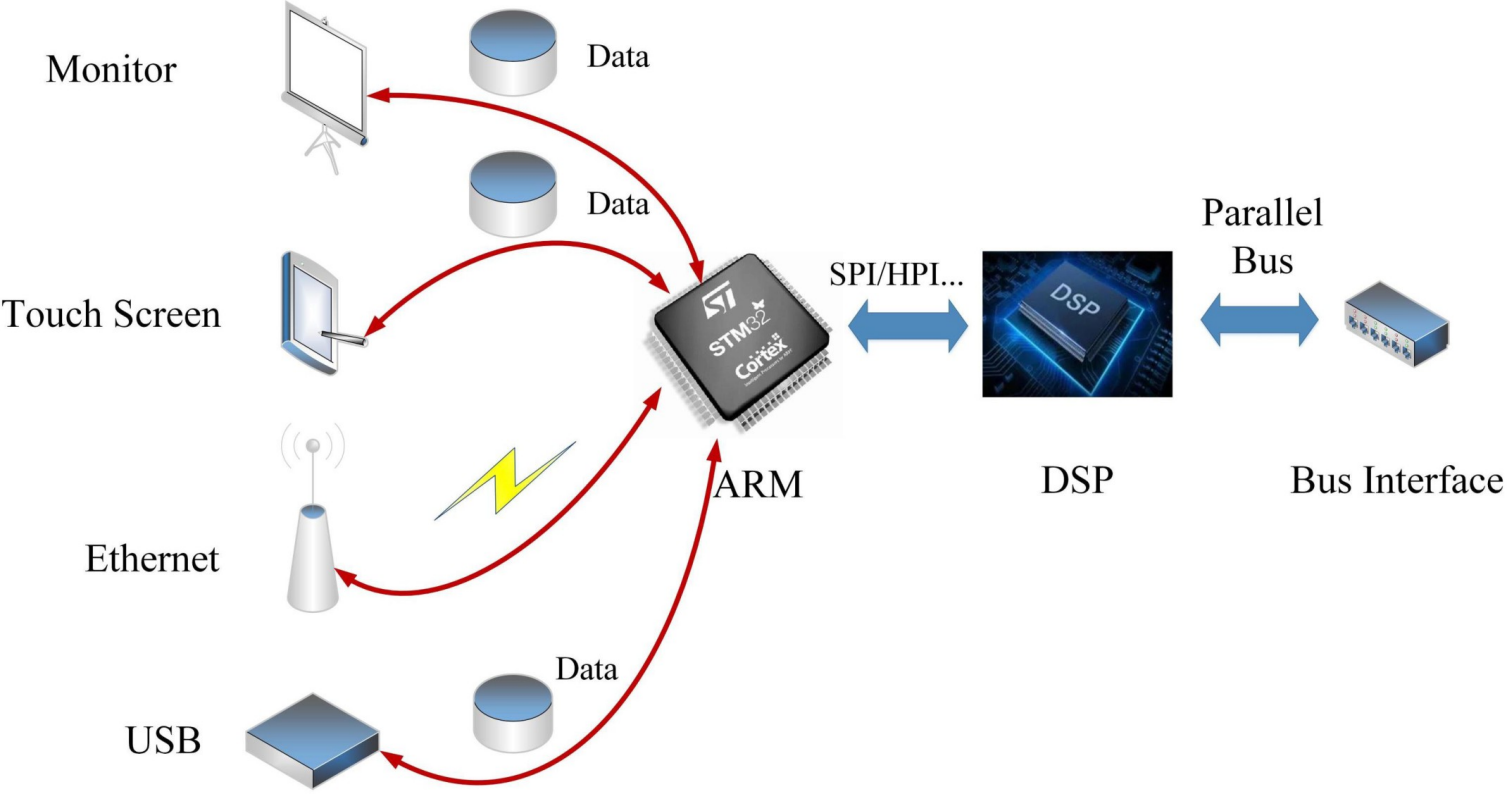
"ARM+DSP" basic architecture.

Therefore, the proposed "ARM+DSP" dual-chip architecture can effectively improve the operation efficiency of the pole mill and speed up the data exchange between the equipment and the monitoring platform. The basic structure of "ARM+DSP" is shown in Fig 3, in which the two control units rely on the HPI (Host Port Interface) interface of the DSP for communication, and can use SPI (Serial Peripheral Interface), IIC (Inter-Integrated Circuit) and other communication protocols. ARM cores typically control the peripheral interfaces throughout the system, relying on drivers on the OS layer to control the core [9], while the DSP mainly performs algorithm acceleration and controls the data storage of the whole system. Also, in order to keep the consistency of resource management on the chip, try to avoid the DSP to access the peripherals [9]. During the system design, it is necessary to separate the memory used by ARM and DSP with strictly physical addresses, as well as to reserve a part of the memory space for interaction to ensure that data transfer is not distorted.

Therefore, this paper applies the "ARM+DSP" dual-chip architecture to the battery wafer production control system, and designs a special battery wafer production control system based on the "ARM+DSP" dual-chip. The dual-chip architecture separates the main drive unit from the winding control unit at the physical layer, and incorporates a deflection correction system to realize the self-correction of wafer deflection. In addition, the Fuzzy PID control strategy is applied to the tension control and deflection control, which improves the control accuracy and robustness compared with the traditional PID control. Finally, the communication protocol, tension module and deflection correction module are experimentally tested.

Our paper is structured as follows: Section 2 summarizes the research work of others in control systems, deflection systems and tension systems. Section 3 analyzes the dual-chip control architecture, the deflection control, and the tension control. Section 4 illustrates the effectiveness of fuzzy PID in the application of deflection control and tension control. Section 5 describes the digital control system and related circuit optimization design methods. Section 6 describes the communication bus test and the experimental test of the deflection control circuit and the tension control circuit. Section 7 summarizes the shortcomings of existing work and provides an outlook for future work. Section 8 describes the contributions of our research.

## Literature review

### Embedded control system

Currently, for industrial sites, many companies use PLC as the main controller and inverters as drive modules. Some common condition monitoring devices are included [10,11]. Such a design, although simple and anti-interference, still has the following problems:

- Less efficient operation than dedicated controllers and poor programming flexibility [11],
- The data processing and transmission method is single and poor in real time.

To improve the efficiency of mechanical equipment, many scholars have used high-performance embedded chips or data processing chips to control lithium equipment [12–14]. Zhao *et al*. [12], proposed the application of embedded technology to design the control system, using an isolated power supply to ensure stable circuit operation and designing relevant circuits to control the connected sensors, inverters, etc. Song *et al*. [13], adopted STM32 as the core controller and designed circuits for switching and analog quantities, and the rolling accuracy was improved to ±0.003 mm. In addition, since the rolling process mainly relies on the driving role of motors, and high-performance embedded platforms have been widely used in motor control [15–20]. Among them, some scholars have used DSP data processing capability to control AC motors [17–20]. Fengcan Zhang *et al*. [20], employed an STM32 control chip to control AC servo motors and optimized the motor control accuracy. The traditional embedded control method is a single chip for control, which inevitably generates some electromagnetic interference, and despite the use of some anti-interference measures, it still cannot be completely eliminated, so some scholars have explored the advantages of dual-chip architecture and achieved results in some fields.

The dual-chip architecture separates the parts that are prone to interfere with each other, which can effectively reduce noise and improve the resolution of the sensor [21], at the same time, Brice.R *et al*. [22], used a model reduction method to analyze the heat dissipation problem of the dual-chip architecture and experimentally verified that the dual-chip architecture can reduce the thermal network creation time by 80%. Therefore, the dual-chip architecture has stronger thermal and immunity capabilities and is more suitable for high-speed mechanical platforms. Dual-chip architectures have been used in temperature control [23], image processing [24], motor control [25,26], RFID(Radio Frequency Identification) temperature measurement [27,28] and prediction control [29], where scholars have divided the modules through the physical layer to ensure stable device operation. In addition, Chung *et al*. [30], applied a dual-chip power management system to the field of photovoltaic power generation and improved the power conversion efficiency to 97.2%.

The dual-core control system of "ARM+DSP" can fully utilize the advantages of digital signal processing to realize data operation. Jiang Han *et al*. [31], proposed a reconfigurable embedded CNC (Computer Numerical Control) machine tool platform based on the hardware architecture of "ARM+DSP", where ARM implements human-machine interaction and interface management and DSP accomplishes complex mathematical operations and real-time tasks. The correctness and effectiveness of the dual chip architecture are verified by taking the six axis bedridden rolling machine as the experimental platform. In addition, the dual-chip architecture of "ARM+DSP" has been applied to cutting platform control [32], sorting system control [33] and power quality detection [34]. Rasesh.D *et al*. [35] used an ARM Cortex A9 processor and FPGA to form a SoC dual-core control unit, where the programmable unit implements the deterministic functions of the power system, the processor unit implements corrective tasks based on field feedback.

The traditional single-chip control system architecture can no longer be applied in high-speed rolling mills. Therefore, according to the mechanical structure of the mill and the operating characteristics of the rolling motor, this paper designs a three-unit integrated digital system based on STM32F407 and DSPTMS320C6713 with dual-chip control, separating the drive and unwinding units. And simulates and analyzes the robustness of the Fuzzy PID strategy for deflection control and tension control. Finally, experimental tests are conducted to verify the effectiveness of the "ARM+DSP" architecture in the pole mill by testing the dual-chip architecture model communication bus and the accuracy of controlled parameters acquisition.

### Deflection system

When the electrode mill is running smoothly, the electrode strip is running fast on the drive roller, but due to the bending and deformation of the electrode strip itself or the influencing factors of the lithium mill equipment and operation process, it will cause the runaway of the lithium battery electrode strip on top of the drive roller. This will not only affect the winding and unwinding of the poles, but may also cause damage to the mechanical equipment in the winding and unwinding section. Therefore, a deflection correction system is needed to solve the problem of run-out of the poles and to keep the poles running in the correct direction in the production line [36].

The main role of the deflection correction system in the smooth rolling process of the poles is as follows:

- Avoid the runaway of the pole piece during the production process and ensure the smooth running of the pole piece on the main roll and drive roll,
- Smooth winding of the pole piece, avoiding folding or wrinkling of the pole piece during the winding process,
- Make the traction force of the pole piece belt parallel to the center line of the drive roller to ensure the uniform tension of the pole piece in the winding and unwinding.

The research of the guiding system originated from European countries. As the demand for deflection systems gradually increased, many research and development enterprises of deflection controllers emerged, such as ST (Italy), FIFE (USA) and BST (Germany), which have widely applied deflection systems in metallurgy, packaging and textile industries. With the gradual increase in the demand for deflection correction, many scholars have analyzed the causes of strip structure runout and also proposed corresponding solutions. You *et al*. [36], the roll design with the best deviation correction ability is designed through the control variable method, and the effectiveness of the strip roll model is verified combined with the PID control method. Image acquisition methods are also gradually used in the design of correction systems [37,38]. However, due to the complex industrial site environment, the optical path information acquisition system error is large. Therefore, this paper adopts a dual-chip control architecture, combined with Fuzzy PID control algorithm and equipped with fieldbus to control the deflection system.

### Tension system

During the winding and unwinding process of the mill, the size and stability of the tension of the pole strip are crucial process indicators. Also tension control is one of the difficult points that affects whether the pole strip can be rolled smoothly [39]. The specific role of tension in the pole rolling process is as follows:

- Maintain the straightness of the lithium battery pole strip to avoid waves caused by uneven extension of the pole strip,
- Smooth and stable rolling of the polar strip to avoid runaway of the polar strip during winding and unwinding,
- Adjust the thickness of the lithium battery electrode strip to meet the set thickness requirements,
- Reduce the deformation resistance of the pole strip and fine-tune the rolling force to reduce energy and material losses.

Tension control for take-up and take-down strips was initially achieved by conventional simulators with motors. Due to the lack of precise detection feedback devices, the production accuracy of the product was low. With the performance of the inspection system and computer processing power gradually improved, the rolling process and control strategy gradually optimized. Tension control methods are also becoming more and more abundant, the traditional simulator is replaced by the existing high-precision sensors and controllers, through the output control signal or adjust the current to complete the tension regulation and control [40].

Many scholars have studied tension control systems, Ashour H *et al*. [40], designed a PLC and HMI (Human Machine Interface) based variable speed AC (Alternating Current) drive hardware system for paper curling, which calculates the reference torque and angular velocity by counting the number of pulses at the nip roll through an encoder. Shokhin *et al*. [41], investigated the tension detection system for the winding mechanism of the pole mill, and realized real-time tension monitoring using tension sensors. Therefore, this paper uses dual-chip architecture to control the winding and unwinding mechanism.

The main existing control methods are robust control, adaptive control, fuzzy control, neural network, and PID control. Among them, robust control has good performance in combating the disturbances within the system and the external environment, but its control accuracy is slightly lower than other methods. Adaptive control has good performance in coping with external random disturbances and hysteresis response method of mechanical equipment, but the hysteresis of closed-loop control of mill tension is not high, and the disturbance is not particularly strong in the stable control process. PID control method is simple and easy to implement and widely used in industry, with no need to establish accurate mathematical models and excellent anti-interference fuzzy algorithm, can be well applied to the closed-loop control process of rolling mills. Therefore, this paper uses fuzzy theory optimized PID control method to control the tension system.

## Theoretical analysis

### Dual-chip architecture

The ARM architecture is a 32-bit RISC (Reduced Instruction Set Computer) processor architecture, while the DSP is a SIMD (Single Instruction Multiple Data) processor architecture. The STM32F407 used in this paper is an ARM branch architecture based on the Cortex-M3 core with a Harvard architecture and a three-stage pipeline selected for microcontroller applications with added branch prediction. The TMS320C6713 is a 32-bit high-speed floating-point DSP with a Harvard architecture. Therefore, the hardware architecture can be designed in combination.

**Fig 4:**
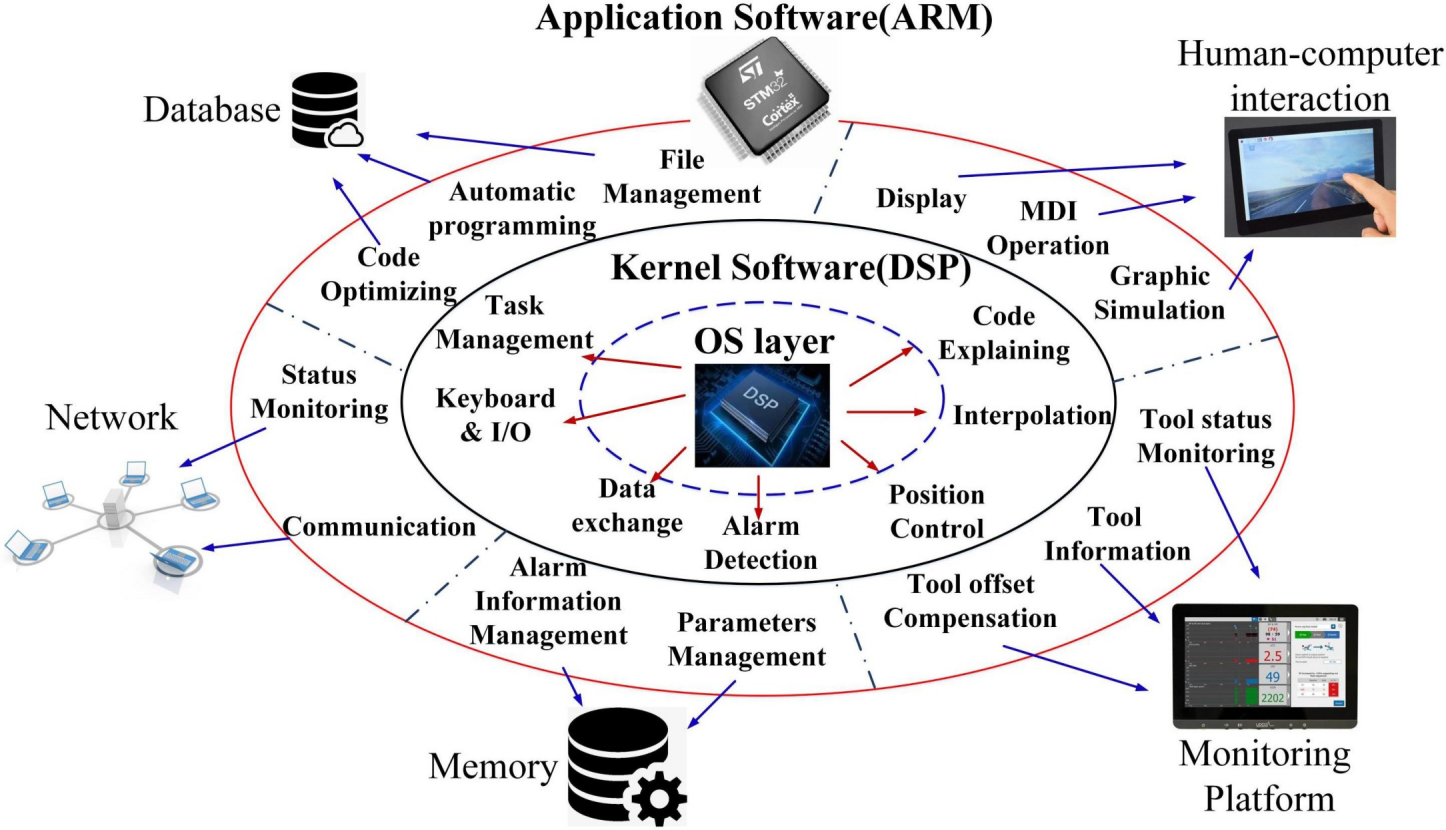
"ARM+DSP" system division.

The dual-chip architecture system division of labor is shown in Fig 4. DSP generally performs complex mathematical operations and real-time tasks, including position control, interpolation calculation and programmable controller control; STM32 mainly performs human-machine interaction and interface management, such as code programming, parameter setting and status display.

The operation of the pole mill includes multi-motor asynchronous operation, tension size mediation, roll slit mediation, deflection mediation and other processes. The dual-chip architecture enables the division of modules with different functions, giving full play to the control capability of ARM and the data processing capability of DSP, and improving the operating efficiency of the mill. Data transfer between dual CPUs can be done through serial, SPI, dual-port RAM, CAN and Ethernet, etc. STM32 and DSP often use SPI protocol to communicate with external chips. SPI communication protocol adopts frame structure, which is a synchronous serial communication interface technology [42]. SPI communication protocol has the advantages of supporting full duplex communication, simple communication mode and relatively fast data transmission rate, the disadvantage is that there is no specified flow control and no response mechanism to confirm whether the data is received.

**Fig 5:**
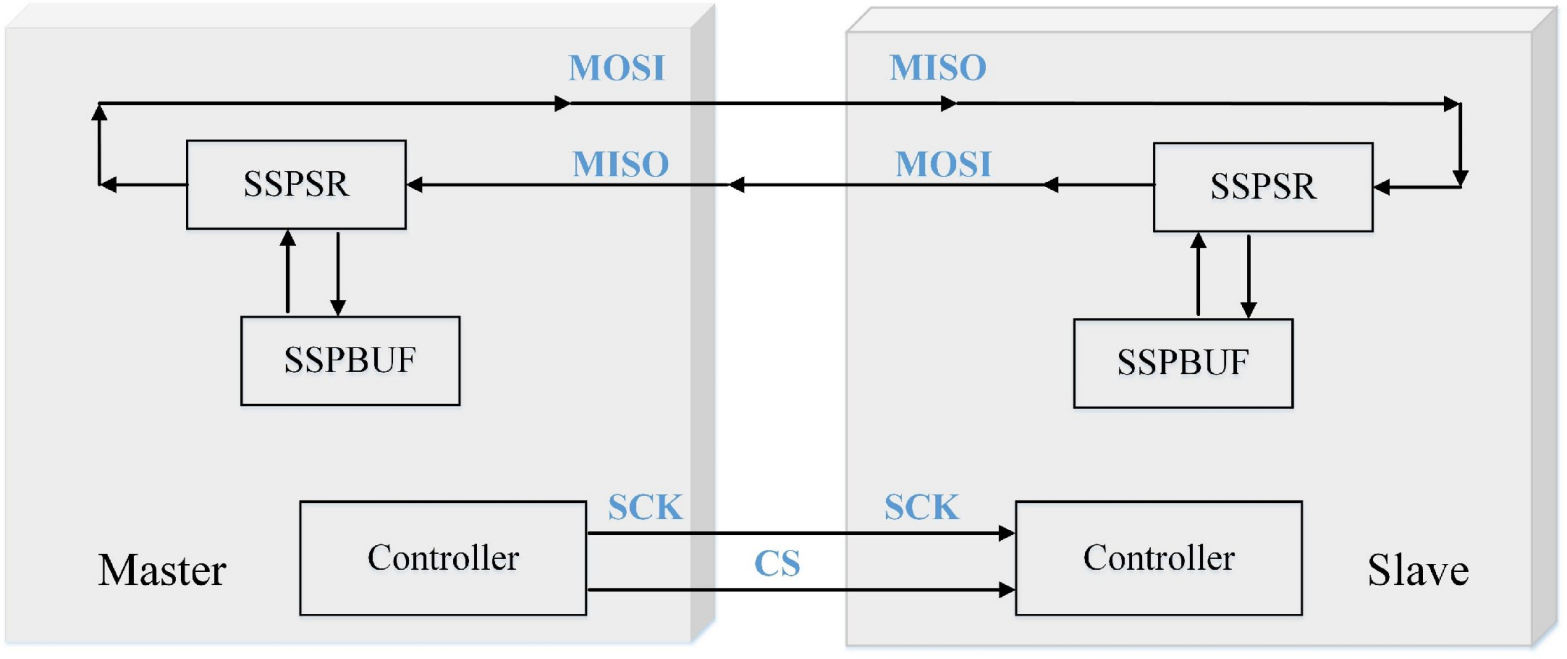
SPI communication mode.

As shown in Fig 5, SPI uses a Master-Slave control method and a timing synchronized data transfer method. The master device generates the clock signal to work synchronously with the slave device [35]. The meaning of each component is: SSPBUF is the internal buffer of SPI device to save the temporary data during transmission. SSPSR is the shift register of SPI device to move the data into or out of SSPBUF. Controller is the CPU controller to configure the SPI bus transmission mode. Usually, only the following four pins need to be configured to achieve SPI communication. SCK: Master device transmits clock signal to Slave device, which controls the timing and rate of data exchange. CS: Master device chip selects Slave device. MOSI: Master device data output, Slave device data input. MISO: Master device data input, Slave device data output.

There are four operating modes of the SPI [35]. The clock polarity CPOL is used to configure which state the SCK level is idle or active, and the clock phase CPHA is used to configure at which edge the data is sampled [43].

**Fig 6:**
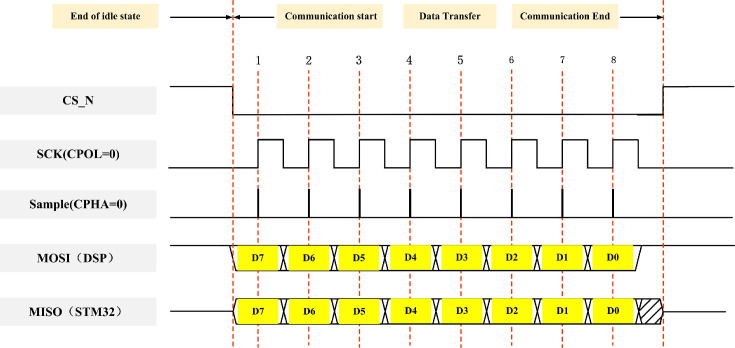
SPI Mode 0 timing diagram.

This paper uses mode 0 operation as shown in Fig 6. The DSP is the Master device, sending data, and the STM32 is the Slave device, receiving data. When CS_N is low, it is in the data transmission state. CPOL = 0, which means it is in the idle state when SCK = 0, so the valid state is when SCK is high. CPHA = 0, which means the data sampling is at the first edge, and the data is sent at the second edge.

### Deflection system

In the actual pole rolling process, the complex structure of machinery and equipment, the large number of actuators in the control system and the influence of external uncertainties can lead to a certain degree of deflection of the pole strip [44]. Among the main factors that can affect the polar strip offset are the following:

- Influence of the geometry of the guide roller,
- Uneven tension distribution due to wave edge,
- The axis between each guide roller is not parallel.

**Fig 7:**
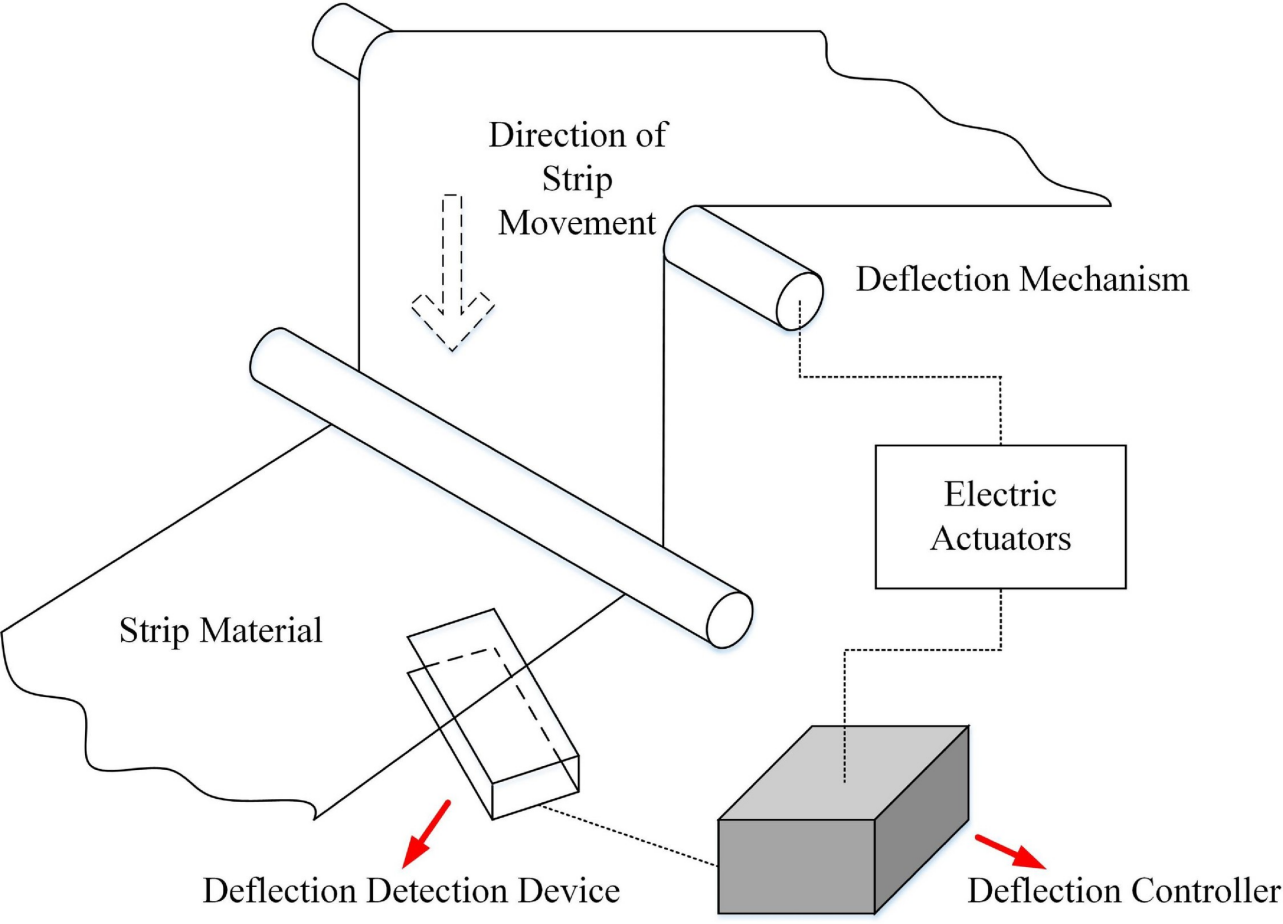
Structure diagram of correction system.

In the winding and unwinding part of lithium battery cells, it is necessary to ensure that the cells are accurately rolled and pressed by the rollers during unwinding, and that the edges are neatly wound by the air expansion shaft during winding. The structure of the pole strip correction system is shown in Fig 7. The pole strip correction system is mainly composed of four parts: deflection detection device, electric actuator, information feedback device and deflection controller. The deviation correction detection device uses the photoelectric sensor installed on one side of the pole strip as the reference. When the pole strip is offset, the detection device will measure the offset distance and angle of the edge of the pole strip to determine the offset degree of the pole strip. The deflection controller determines the control strategy after analysis and processing, and controls the drive motor to drive the deflection mechanical equipment to realize the pole piece belt deflection. When the offset distance of the pole strip is corrected to return to the set value, the electric actuator action is stopped so that the pole strip operates stably within the set offset range [36].

**Fig 8:**
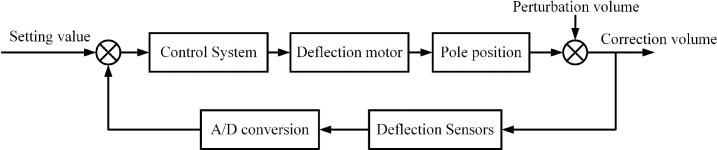
Corrective flow diagram.

In summary, the flow of pole piece correction is shown in Fig 8. The pole strip deflection operation process is a typical closed-loop feedback control link. The commonly used correction detection device is photoelectric or ultrasonic type correction sensor, which collects the position information of the edge of the pole piece in real time and transmits the signal to the main control system. The electric actuator adopts servo driver to drive the servo motor, which drives the screw guide and push rod to move to complete the correction of the offset distance.

### Tension system

The tension control method can be mainly divided into open-loop control and closed-loop control according to the process, and the open-loop control lacks the corresponding feedback link, so the anti-interference ability and rolling accuracy are slightly inadequate [45]. The closed-loop control adds a feedback link to the tension, so that the input and output signals of the whole system can be contrasted and deviations can be eliminated, which can significantly improve the accuracy and anti-interference ability.

**Fig 9:**
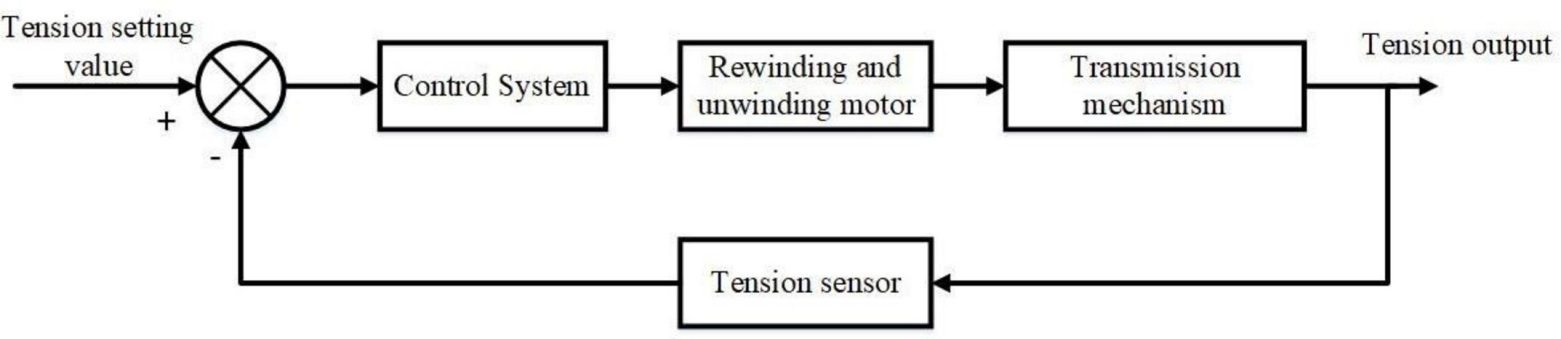
Tension closed-loop control flow chart.

As can be seen from Fig 9, the real-time tension value collected by the tension sensor is calculated by the deviation from the set tension value, so that the control system drives the motor to drive the mechanical drive mechanism to change the speed of the pole piece belt drive. Then the tension sensor compares the tension value with the set value, forming a continuous and accurate closed-loop tension control system.

## Control strategy optimization study

### Research on corrective control methods

#### Fuzzy PID control study for correction of deflection

The deviation correction fuzzy control needs to divide the basic universe of input and output parameters and transform it into the fuzzy universe of fuzzy reasoning operation combined with Q-Factors (Quantization Factors). On the basis of the actual operating parameters of the rolling mill, this paper selects appropriate quantitative factors to realize the transformation of fuzzy universe of control parameters, as described in Table 1. *e* represents the pole position deviation, *e*_*c*_ represents the deviation rate of change, ΔK_P_, ΔK_I_ and ΔK_D_, represent the proportional, integral and differential coefficients, respectively.

**Table 1:** Fuzzy theory and quantitative factors of rectifying deviation.

Parameters	Fuzzy Theory Domains	Q-Factors	Basic Theory Domains
*e*	[-6, -4, -2, 0, 2, 4, 6]	1/2	[-12, 12]
*e*_*c*_	[-6, -4, -2, 0, 2, 4, 6]	1/2	[-12, 12]
ΔK_P_	[-6, -4, -2, 0, 2, 4, 6]	3	[-2, 2]
ΔK_I_	[-3, -2, -1, 0, 1, 2, 3]	3	[-1, 1]
ΔK_D_	[-3, -2, -1, 0, 1, 2, 3]	6	[-0.5, 0.5]

**Fig 10:**
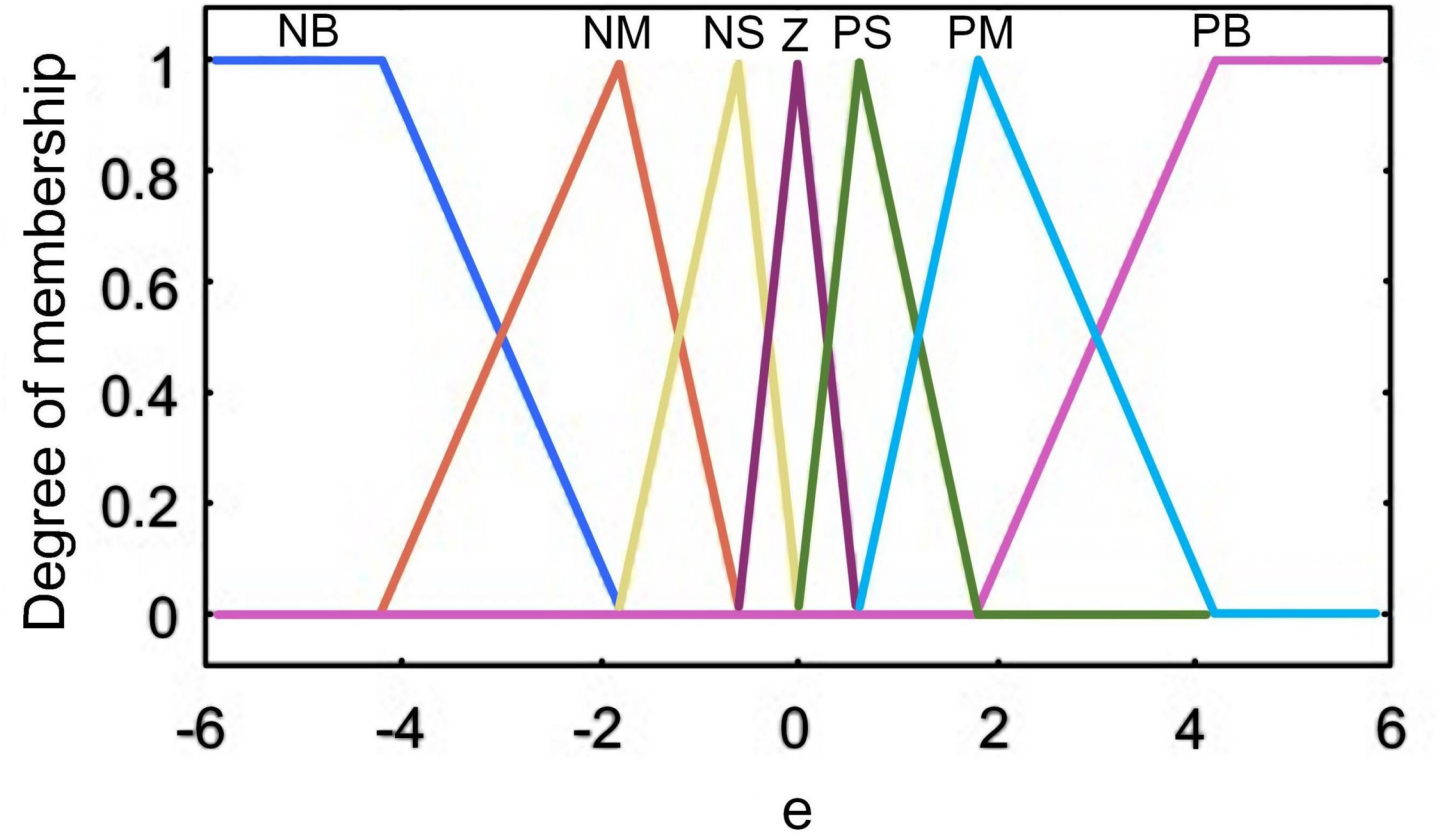
Affiliation function of polar slice correction bias *e*.

According to the characteristics of real-time correction control and the need to achieve fast response of correction, the resolution of the fuzzy set of the affiliation function can be set lower. Taking *e* as an example, the established affiliation function is illustrated in Fig 10. The left and right boundary parts use semi-trapezoidal affiliation function, and the five quantization levels in the middle use triangular affiliation function, which in terms of structure division, the middle part is dense, and the closer it is to both sides, the more sparse it is.

After determining the fuzzy affiliation function, it is necessary to determine the relationship between ΔK_P_, ΔK_I_ and ΔK_D_ in the PID. Considering that the variation of *e* under different operating conditions varies greatly, it is not possible to use expert knowledge for pre-setting, but the following rules can be followed:

- When *e* is in a large range, a larger ΔK_P_ is chosen to make *e* fall rapidly. When *e*_*c*_ is large then it is necessary to avoid overshoot by weakening the effect of the differential link by appropriately reducing ΔK_D_.
- When *e* is in the middle range, ΔK_P_ is increased, ΔK_D_ and ΔK_I_ are decreased to reduce oscillations and static errors. When *e*_*c*_ is centered, ΔK_D_ is taken to be moderate to stabilize the differential link.
- When *e* is in a small range, a smaller ΔK_P_ and a larger ΔK_I_ are chosen. And when *e*_*c*_ is small, the value of ΔK_I_ should be reduced appropriately.

Combined with reference materials and after several simulation tests, the ΔK_P_, ΔK_I_ and ΔK_D_ fuzzy control rules are determined as shown in Table 2.

**Table 2:** Fuzzy control rules table of ΔK_P_、ΔK_I_ and ΔK_D_ for deviation correction.

*e*_*c*_	*e*						
	-6	-4	-2	0	2	4	6
-6	6/-3/1	6/-3/-1	4/-3/-3	4/-2/-3	4/-2/-3	2/0/-2	0/0/1
-4	6/-3/1	4/-3/-1	4/-2/-3	4/-2/-2	2/-1/-3	0/0/-1	-2/0/0
-2	4/-2/0	4/-2/-1	4/-1/-2	2/-1/-2	0/0/-1	-2/1/-1	-4/1/0
0	4/-2/0	2/-1/-1	2/-1/-1	0/0/-1	-2/1/-1	-4/1/-1	-4/2/0
2	4/-1/0	2/-1/0	0/0/0	-2/1/0	-4/1/0	-4/2/0	-4/2/0
4	2/0/3	0/0/-1	-2/1/1	-4/2/1	-4/2/1	-4/3/1	-6/3/3
6	0/0/3	-2/0/2	-4/1/2	-4/2/2	-4/3/1	-6/3/1	-6/3/3

#### Simulation testing

By combining the three transfer functions of detection comparison, motor drive and mechanical drive in the deflection control, the transfer model of the deflection correction system can be obtained as follows:

$$G(s)=K_{L}\times \frac{K_{d}}{T_{m}s^{2}+T_{d}s+1}\times G_{s}(s)=\frac{K_{L}K_{d}}{T_{m}s^{2}+T_{d}s+1}\times e^{-\tau_{0}s}$$ (1)

Where *T*_*m*_, *T*_*d*_ and *K*_*d*_ are constants and are related to the electromagnetic torque of the motor, *K*_*L*_ represents the correction detection link conversion factor.

According to the conversion calculation of the output analog voltage of the correction photoelectric sensor and the position deviation information in the actual correction control, *K*_*L*_ can be obtained as 0.1. According to the mechanical device delay link, *τ*_*0*_ will be to 0.1. Combining the relevant parameters such as armature voltage, rotation angle and potential of the correction motor, the speed and voltage transfer model of the motor can be obtained as followed:

$$G(s)=\frac{3.5}{5s^{2}+0.8s+1}\times e^{-0.1s}$$ (2)

**Fig 11:**
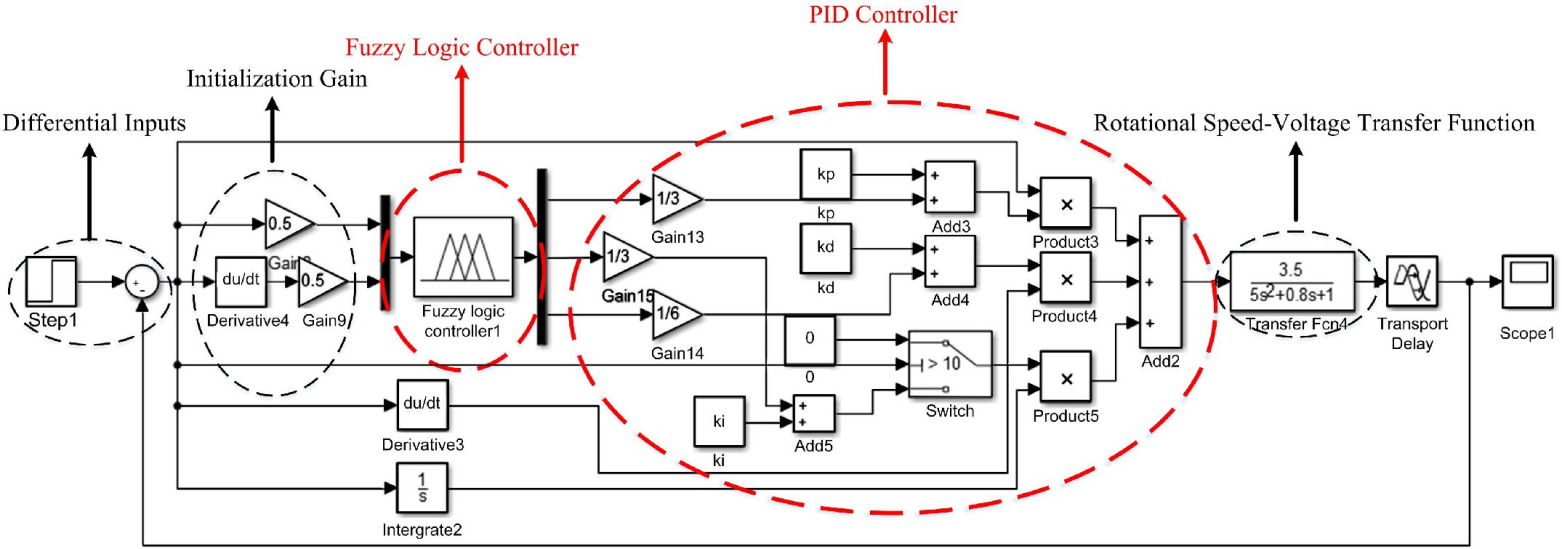
Fuzzy PID correction simulation model.

Fuzzy PID simulation model for correction is shown in Fig 11. The authors in [38] only used the pole position deviation as the fuzzy input. But obviously, the deviation change rate also needs to be considered. This paper takes the difference between the set step signal and the final output signal and the change rate of the difference as the input of the fuzzy controller. The differential signal is passed through two-stage amplifier to initialize the system gain, and then the parameters are introduced into fuzzy logic controller 1. The optimized PID parameters K_P_, K_I_ and K_D_ are output and combined with the speed-voltage transfer function to form a closed-loop feedback control.

**Fig 12:**
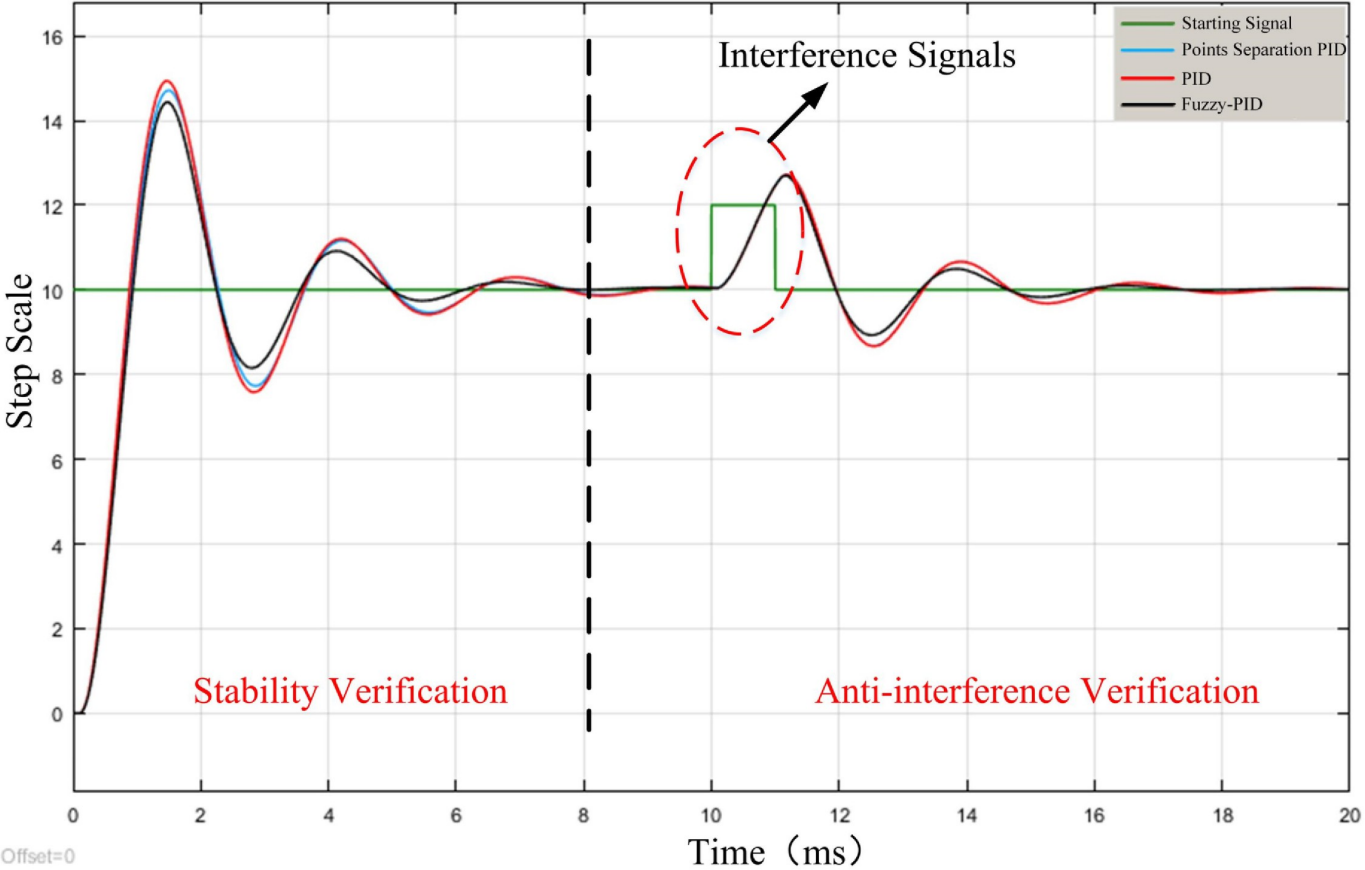
Small interference signal simulation.

**Fig 13:**
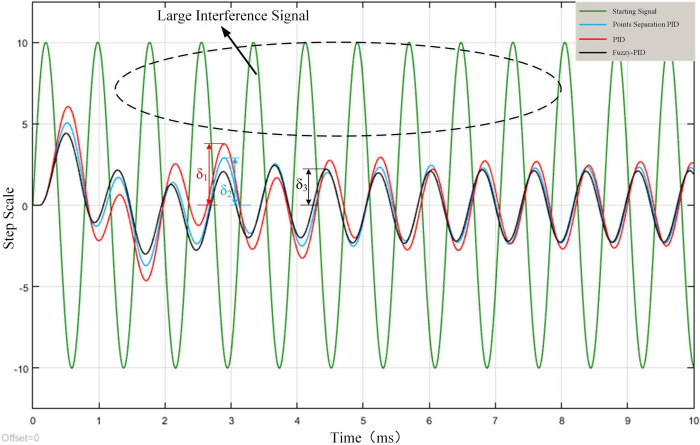
Large interference signal simulation.

The step signal is used as the input signal in the simulation test, and the sinusoidal signal and smaller step signal are used as disturbances. The threshold is set to 5, when the integral link in PID is eliminated. Add PID model, integral separation PID model for experiments, simulation results are shown in Figs 12 and 13.

When the set disturbance signal is small, it will not trigger the integral separation, so the PID and integral separation PID control curves overlap. And it can be observed that the Fuzzy PID has a stronger ability to adapt to the disturbance and can revert to the initial step value and reach the steady state faster. When large sinusoidal disturbance is added, all three methods have certain resistance to the disturbances, and the output waveforms of all three control methods show oscillations with the sinusoidal curve, but the deviation δ_3_ of Fuzzy PID is the smallest, and the deviation δ_2_ and δ_1_ of integral separation PID and PID are larger. Therefore, the Fuzzy PID has a greater advantage in terms of anti-jamming and anti-oscillation.

Under the action of the same control signal, although the PID control response is faster, but the response rate of the three methods is relatively close. In the analysis of the subsequent overshoot oscillation and finally reach the steady state time situation can be seen, the Fuzzy PID effect is optimal.

### Research on tension control methods

#### Fuzzy PID control study for tension of deflection

The unwinding and rewinding tension control methods are the same, so only the unwinding tension Fuzzy PID control is introduced. Combined with the changes of tension in the actual unwinding control process of the polar strip, Q-Factors are selected to complete the theoretical domain transformation of the parameters *e’* and *e*_*c*_*’* and the optimal variation range of ΔK_P_, ΔK_I_ and ΔK_D_ in the PID control process, the specific parameter formulation rules are shown in Table 3. *e’* represents the unwinding tension deviation, *e*_*c*_*’* represents the rate of change of deviation.

**Table 3:** Fuzzy theory and quantitative factors of unwinding tension.

Parameters	Fuzzy Theory Domains	Q-Factors	Basic Theory Domains
*e’*	[-6, -4, -2, 0, 2, 4, 6]	1/2	[-12, 12]
*e*_*c*_ *’*	[-3, -2, -1, 0, 1, 2, 3]	1/2	[-6, 6]
ΔK_P_	[-6, -4, -2, 0, 2, 4, 6]	3	[-2, 2]
ΔK_I_	[-6, -4, -2, 0, 2, 4, 6]	3	[-2, 2]
ΔK_D_	[-6, -4, -2, 0, 2, 4, 6]	6	[-1, 1]

**Fig 14:**
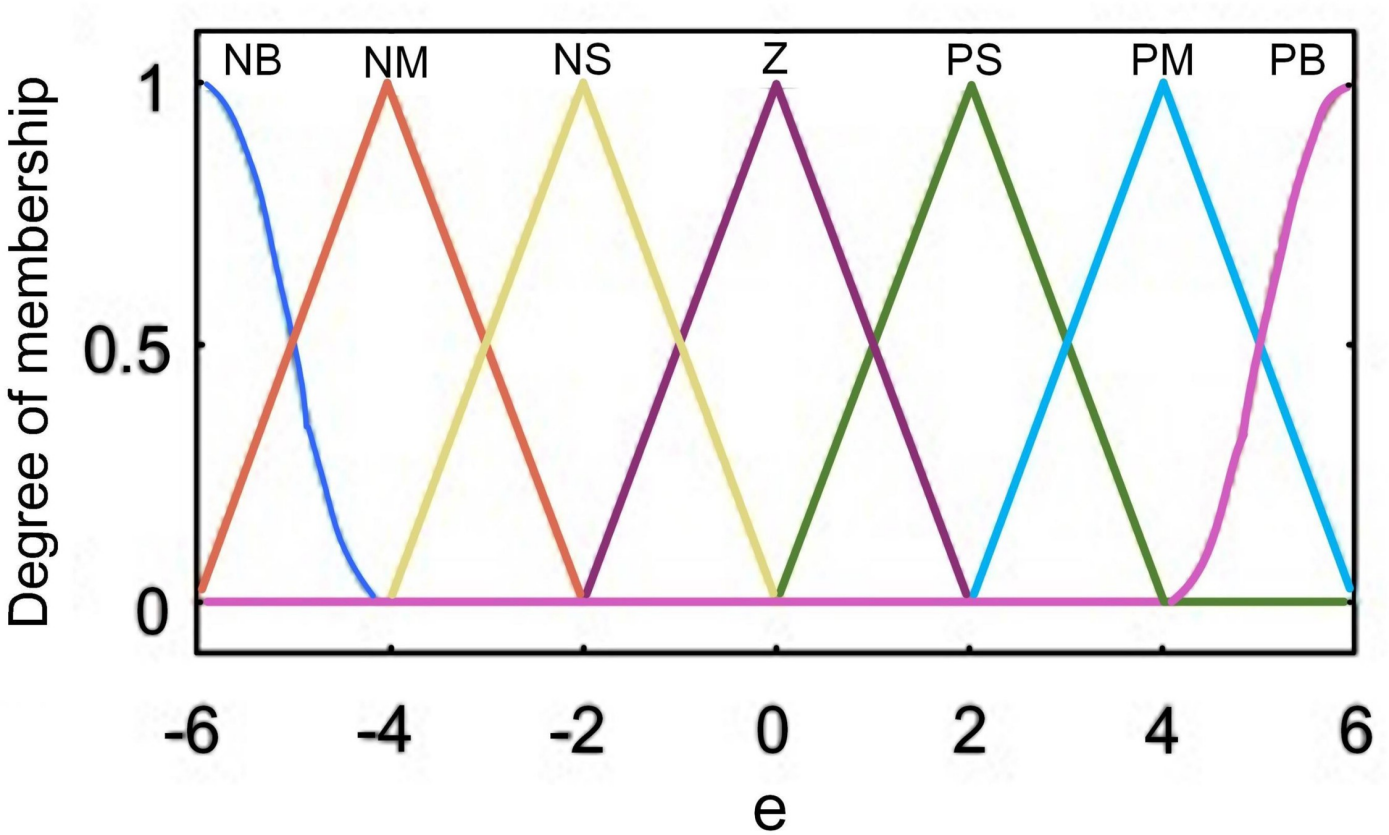
Affiliation function of unwinding tension deviation bias *e*.

Taking the *e’* as an example, the affiliation function of tension control is constructed as in Fig 14. Both input and output parameters use Z-type and inverse Z-type affiliation functions to form the left and right boundaries, so that the affiliation at the boundary can achieve a smooth transition from 0–1 or from 1–0. When the pole mill is running smoothly and the tension is stable in operation without external interference, showing a symmetric structure of left and right. The trigonometric affiliation function is more sensitive than the normal distribution and other affiliation functions when the input changes.

In determining the variation of ΔK_P_, ΔK_I_ and ΔK_D_ parameters during PID control, the following rules need to be followed:

- When the pole mill is in the start-up stage, the unwinding device is disturbed by mechanical parts. The pole piece position is unstable, the pole piece position deviation *e* is large, and the tension deviation *e*^*’*^ has a large fluctuation range. It is necessary to reduce the value of ΔK_P_ and ΔK_I_ appropriately and increase the value of ΔK_D_ to prevent the output integral from saturating.
- When the pole mill is in stable operation, the tension does not change dramatically in a short period of time, *e’* and *e*_*c*_*’* are small, and the ΔK_P_ and ΔK_I_ is gradually increased to improve the stability of the control.
- In the process of pole piece belt transmission, the *e*_*c*_ is small, but there is a sudden change of the *e*. At this time, the *e*^*’*^ and *e*_*c*_*’* change suddenly in a short time to form a spike pulse. In this case, ΔK_I_ should be increased appropriately and ΔK_P_ should be reduced, while ΔK_D_ should be taken as a small value.

Combining expert knowledge and multiple experimental simulations to obtain the tension fuzzy rule is shown in Table 4.

**Table 4:** Fuzzy control rules table of ΔK_P_、ΔK_I_ and ΔK_D_ for unwinding tension.

*e*_*c*_	*e*						
	-6	-4	-2	0	2	4	6
-3	6/-6/2	6/-6/-2	4/-4/-6	4/-4/-6	2/-2/-6	0/0/-4	6/0/2
-2	6/-6/2	6/-6/-6	4/4/-6	2/-2/-4	2/-2/-4	0/0/-2	-2/0/0
-1	4/-6/-6	4/-4/-2	4/-2/-4	2/-2/-2	0/0/0	-2/4/2	-2/4/2
0	4/-4/0	4/-4/-4	2/-2/-2	0/0/0	-2/2/-2	-4/4/-2	-4/4/0
1	2/0/6	2/-2/2	0/0/0	-2/2/0	-2/2/0	-4/4/4	-4/6/0
2	2/0/6	0/0/4	-2/2/4	-4/2/4	-4/4/2	-4/6/2	-6/6/6
3	0/0/6	0/0/4	-4/2/2	-4/4/4	-4/4/2	-6/6/6	-6/6/6

#### Simulation testing

By analyzing the causes of tension generation, the unwinding tension is obtained as:

$$T=\frac{ES}{L}\int (v_{main}-v)dt$$ (3)

Where *v*_*main*_ for the mill main roll running line speed, *v* for the release roll pole sheet with the exit line speed, *E* for the elastic modulus, *S* for the Polar strip cross-section area, *L* for the length of polar strip.

Deriving Eq (3) yields:

$$\frac{dT}{dt}=\frac{ES}{L}(v_{main}-v)$$ (4)

**Fig 15:**
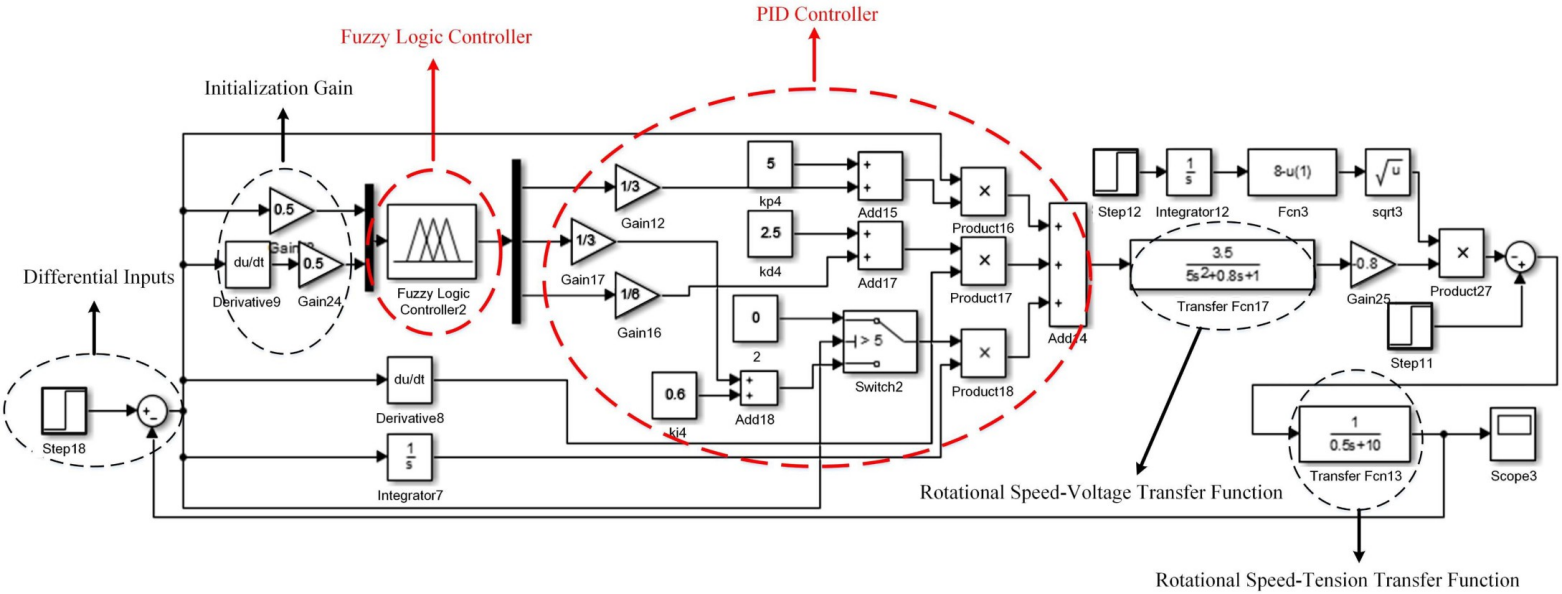
Fuzzy PID tension simulation model.

Under the premise of stable and reliable torque, *v* is only related to the speed of the unwinding motor and the roll diameter of the unwinding roll. By appropriately selecting the signal conversion scale factor, we can get motor-speed voltage transfer function is $\frac{3.5}{5s^{2}+0.8s+1}$, speed-tension transfer function is $\frac{1}{0.5s+1}$. The authors in [40] only considered the influence of motor speed difference on tension. After analysis, we determine the influence of deviation correction control on tension control, and add the influence relationship into fuzzy knowledge base. The tension Fuzzy PID simulation model is shown in Fig 15, different from the deviation correction control model, the feedback output is not only determined by motor speed and voltage, but also the relationship between motor speed and tension. This paper takes the difference between the step signal and the final output as the input, and uses the fuzzy controller to optimize the three parameters of PID. *Step11* and *Step12* are the set linear speed of the pole strip through the main roll.

**Fig 16:**
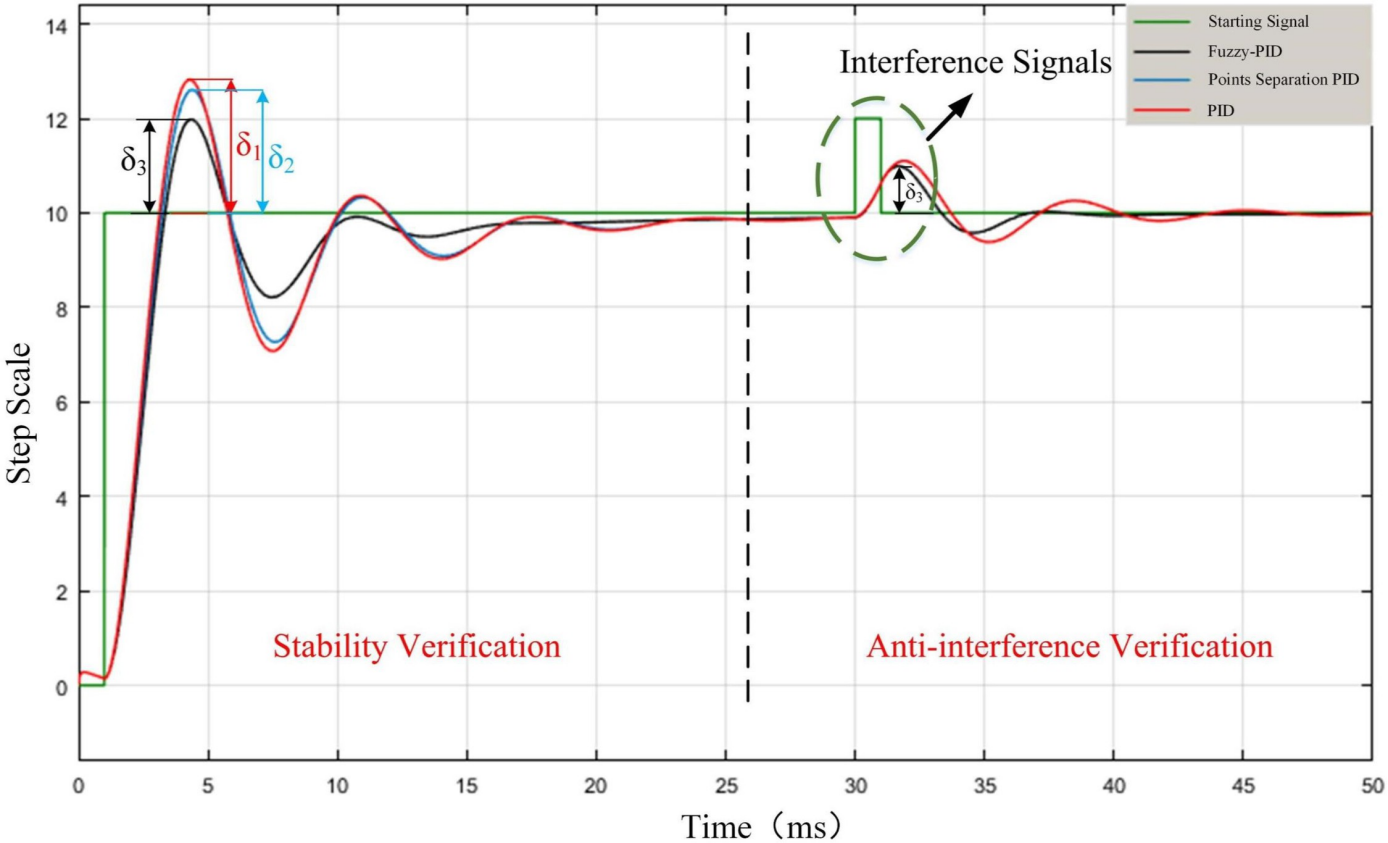
Interference signal simulation.

The initial values of three coefficients in PID control *K*_*P*_*’*, *K*_*I*_*’* and *K*_*D*_*’* are taken as 5, 0.6 and 2.5, and the threshold value of the integration separation principle is set as 5. Adding PID model, integration separation PID model for experiment, the interference step signal amplitude is 10, and the simulation results are shown in Fig 16. *Step11*, *Step12* and *Step16* differential input system, therefore, at the beginning of the signal, there is a weak oscillation in the same direction as the step signal.

The deviation amount δ_1_ of PID is the largest, and the deviation amount δ_3_ of Fuzzy PID is the smallest. The two curves corresponding to the PID and the integral separation PID do not overlap because the step value is greater than the set threshold value that causes the integral separation. After putting in disturbance, δ_1_ is obviously larger than δ_3_, and the final time to reach steady state is much larger than that of Fuzzy PID. Therefore, the stability of the Fuzzy PID control method is optimal.

## Digital control system optimization design

### Overall design plan

System integration is the tailoring of numerous technologies and products according to the user’s needs, the appropriate and reasonable selection of relevant technologies and strategies. Then selecting and configuring the best software and hardware products and resources. Finally the combination into a complete and integrated solution that can solve the application needs of the device [46,47].

Pole mill is a transmission equipment controlled by multiple motors in complex factory environment [48]. For multi-motor control and harsh production environments, the following problems are generally encountered in the design of their digital hardware system integration [45]:

- The control circuit of the motor generates high frequency, RF, spikes and other multi-coupled interference signals,
- Complex control sequence and control logic,
- Large electricity requirements and unstable power supply.

**Fig 17:**
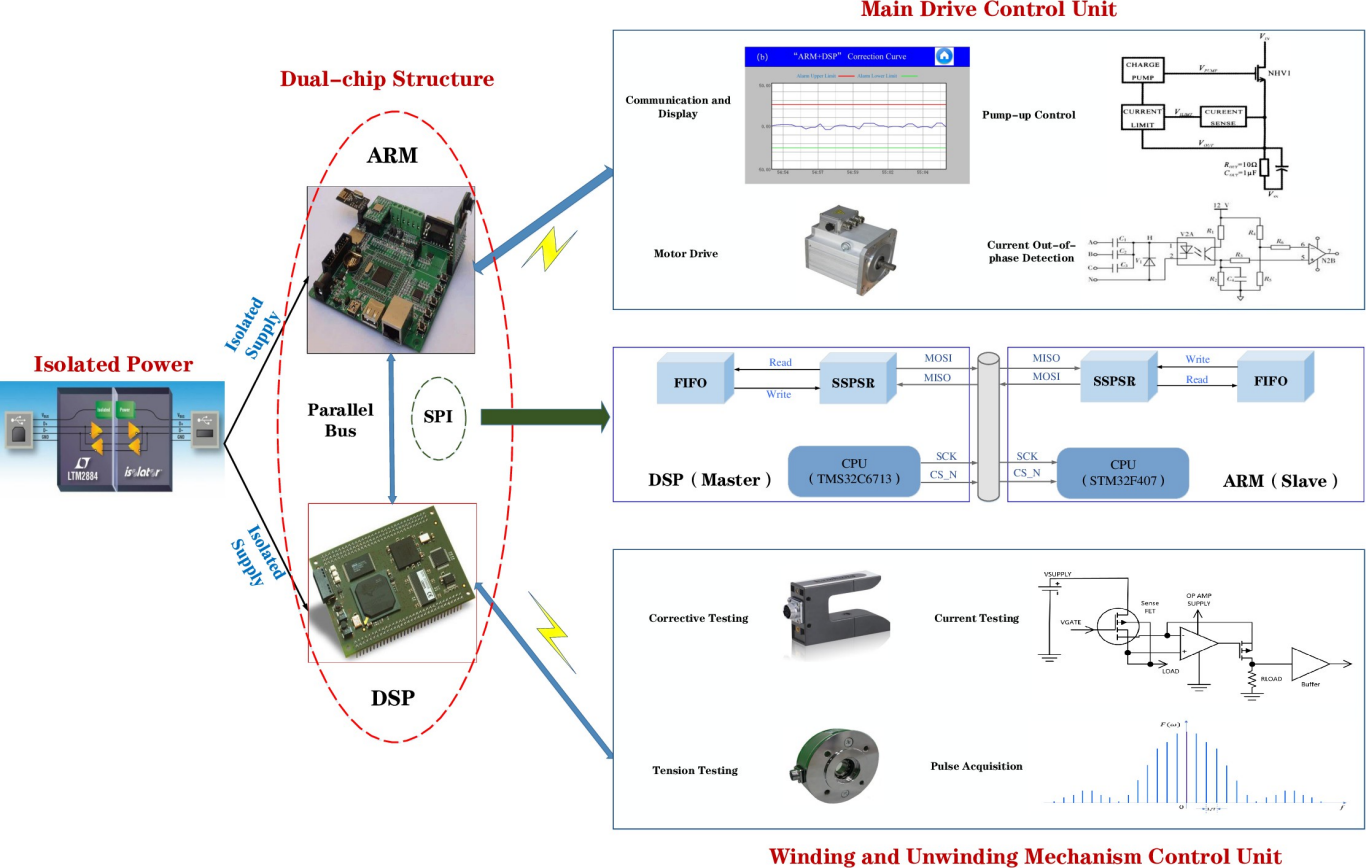
Hardware architecture block diagram.

To overcome the interference between multiple signals, a three-unit cascade architecture is used, as shown in Fig 17. The main control unit adopts the dual-chip architecture of "ARM+DSP", in which ARM mainly controls the asynchronous motor drive and realizes the real-time display of data. DSP is responsible for the acquisition and data processing of various detection quantities. The DSP (Master) sends data to the STM32 (Slave) via the MOSI signal line, along with the clock signal SCK and the chip select signal CS_N. The data to be sent is stored in the FIFO and sent via the shift register SSFSR. The STM32 receives data synchronously and stores it in a FIFO. To overcome spikes and other interference, an isolated power supply is used.

### Control circuit optimization design

#### Deflection control module

**Fig 18:**
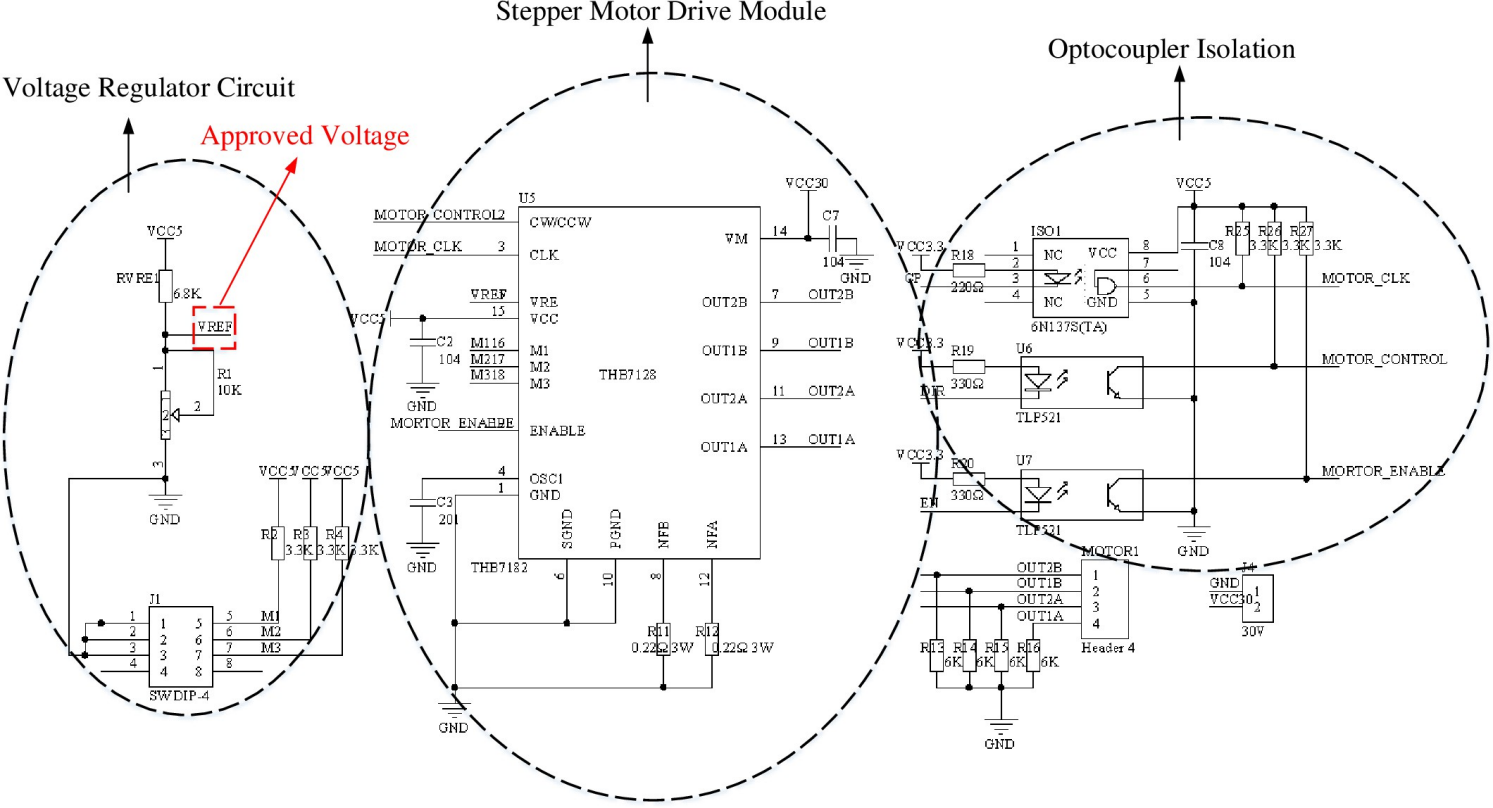
Deflection drive circuit.

Authors in [37] adopts the combined driving mode of NPN and PNP triodes, but this mode has limited driving capacity and cannot drive high-power motors. The deviation correction system needs to be driven by 80W stepping motor, so the multi-channel optocoupler driving mode is selected, and the deviation correction driving circuit based on pulse plus direction control mode is designed [38]. The specific circuit is shown in Fig 18.

The voltage regulator circuit adjusts the input voltage to the VREF reference voltage to supply power to the stepper motor driver chip. M1, M2, M3 are subdivision setting signals, which are used to subdivide the stepper driver chip by changing the high and low levels. The most precise subdivision can reach 1 / 128, which are pulse CP, direction DIR and enable EN respectively. The pulse signal should be connected to the CLK of the chip via the high-speed optocoupler 6N137, and the direction signal and enable signal should be connected to the ENABLE pin of the chip via the common optocoupler TLP521. To drive an 80W stepper motor, a drive current of 2.7 A is required. The current setting formula is:

$$I=VREF*\frac{1}{5}*\frac{1}{R_{s}}$$ (5)

Where R_S_ is the external detection resistor, R_1_ can change the value of the reference voltage at any time to adapt to the size of the controlled motor power. Through R_1_ and R_5_ resistor voltage division can be calculated, VREF = 3 V, R_S_ = 0.22 Ω, where R_1_ is a sliding resistor can be changed at any time to adapt the value of the reference voltage to the size of the controlled motor power.

#### Tension detection module

**Fig 19:**
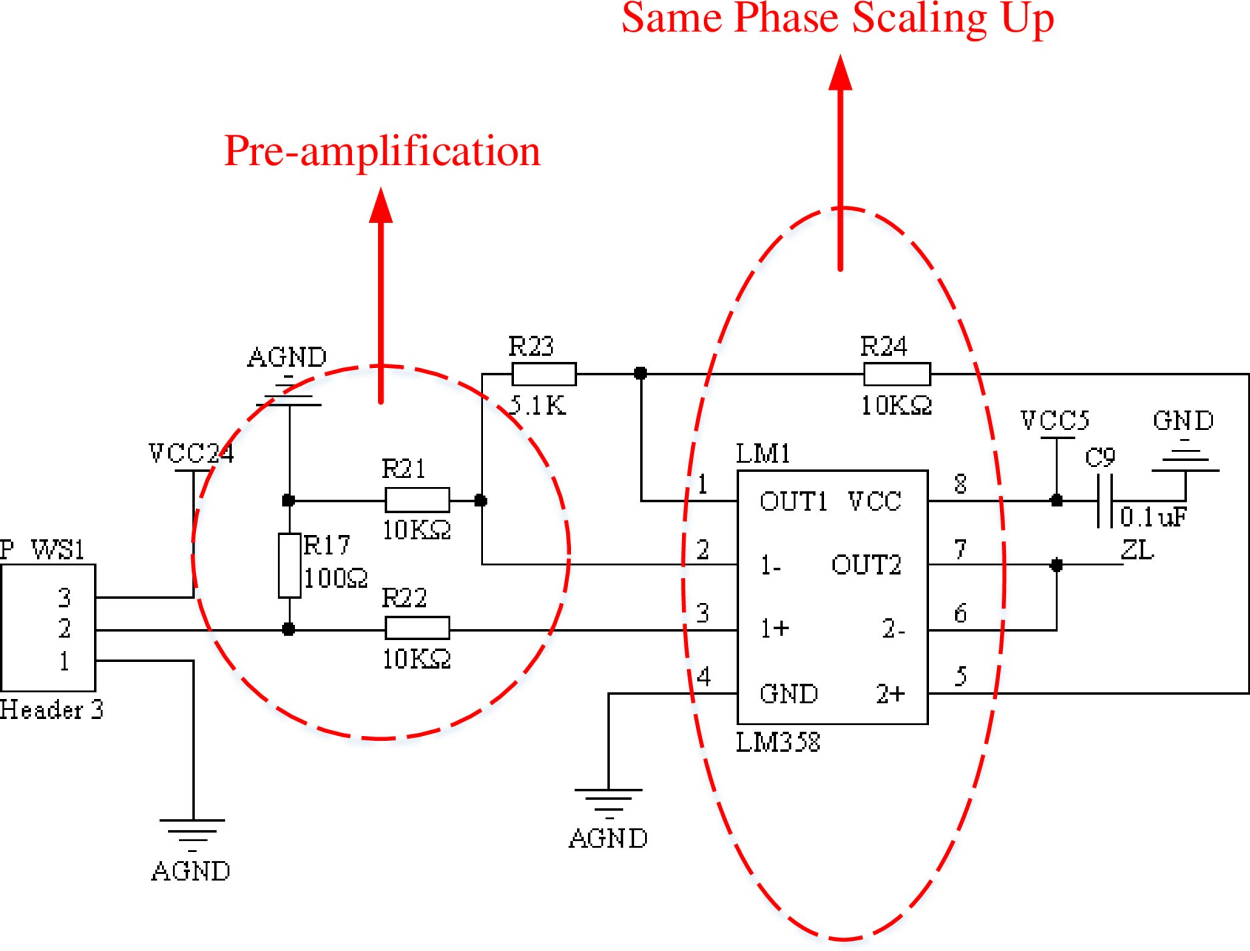
Tension detection circuit.

The tension signal is an analog signal, and most of the analog detection circuits are detected by a single signal source, which cannot be applied to the control field of multi signal input [49]. Considering the strong compatibility of the integrated control system, the tension detection circuit designed in this paper can detect 4-20mA current signal and 0-10V voltage signal at the same time. The design schematic is shown in Fig 19. The input electrical signals of two different types of sensors, 4–20 mA or 0–10 V, are converted into 0–3.3 V voltage signals that can be recognized by the built-in AD converter of the dual control chip.

The two operational amplifiers inside the LM358 are set to amplify the voltage with the in-phase proportional amplification function and the voltage following function, and then the voltage following circuit matches the impedance to play the role of buffering. The tension signal is a current signal of 4–20 mA, which is converted to a voltage signal of 0.4–2 V by means of a resistor. Using an in-phase proportional amplifier circuit to enhance the measurement range. Tension detection signal of 0.6 V to 3 V is transmitted to the DSP through a voltage following circuit.

## Experimental testing

### Communication testing

**Fig 20:**
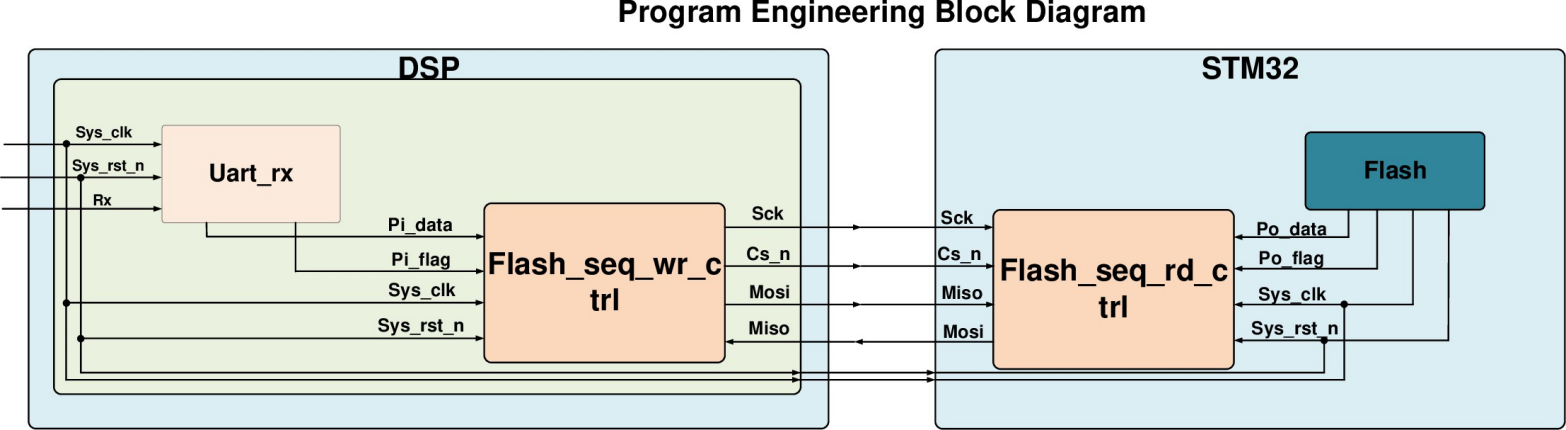
Program engineering block diagram.

**Fig 21:**
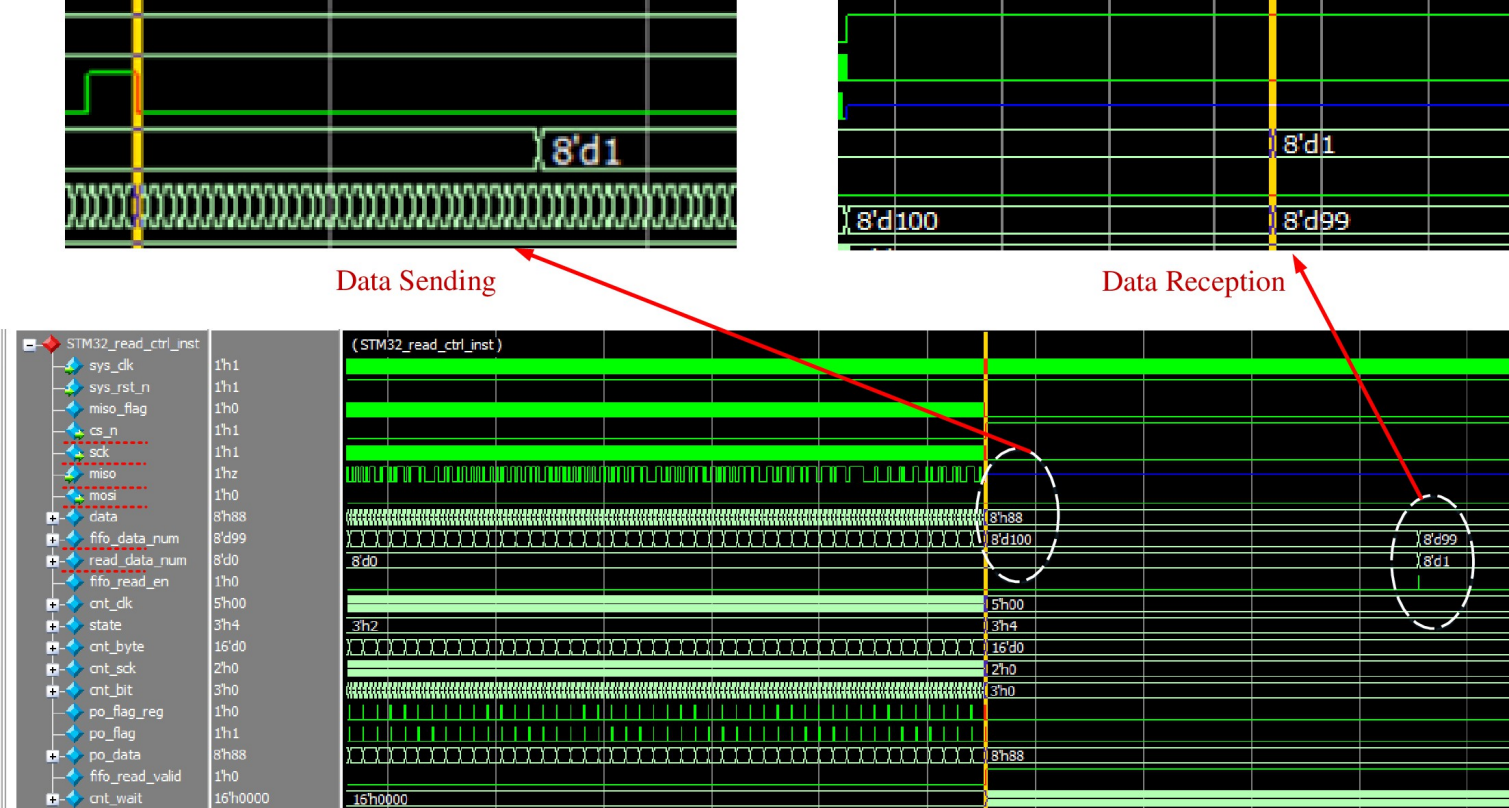
SPI communication simulation waveform.

In order that the parameters obtained after the information acquisition module of DSP and processing can be accurately transferred to the STM32 master control, and the STM32 will realize the control of motor and inverter. The test process ensures that the master and slave are in synchronization. Firstly, the RS-232 interface is used to write 8-bit decimal data to the master. After that, the master receives the upper computer data through the serial port, prepares the SPI bus, sends 1bit preparation signal to the slave, and configures the slave SCK clock signal and CS chip selection signal. The program design follows the modular design idea, and the program engineering block diagram is shown in Fig 20. During SPI communication test, take DSP as the master to be responsible for clock and data output; STM32 is a slave to complete data reception. The STM32 FLASH saves the received data, and the STM32 sends the received data to the DSP again to complete the process of one master and one slave data sending and receiving. The simulation waveform is shown in Fig 21. The STM32 first receives the data sent by the DSP, and when the CS_N chip select signal is high, data reception begins, and due to the communication protocol, the sending and receiving process lags behind Sys_clk by one clock cycle. When FLASH finishes receiving data, it starts sending data to DSP. Through simulation experiments, the following conclusions are drawn:

- The DSP needs to send an SPI ready signal to the STM32 to overcome the answer mechanism without the data acknowledgments bit,
- DSP sends data through MOSI, STM32 receives data through MISO, both keep synchronized,
- DSP and STM32 through the SPI bus communication stability, in the industrial field need to add anti-interference technology to ensure stable data transmission.

### Tension detection and control testing

**Fig 22:**
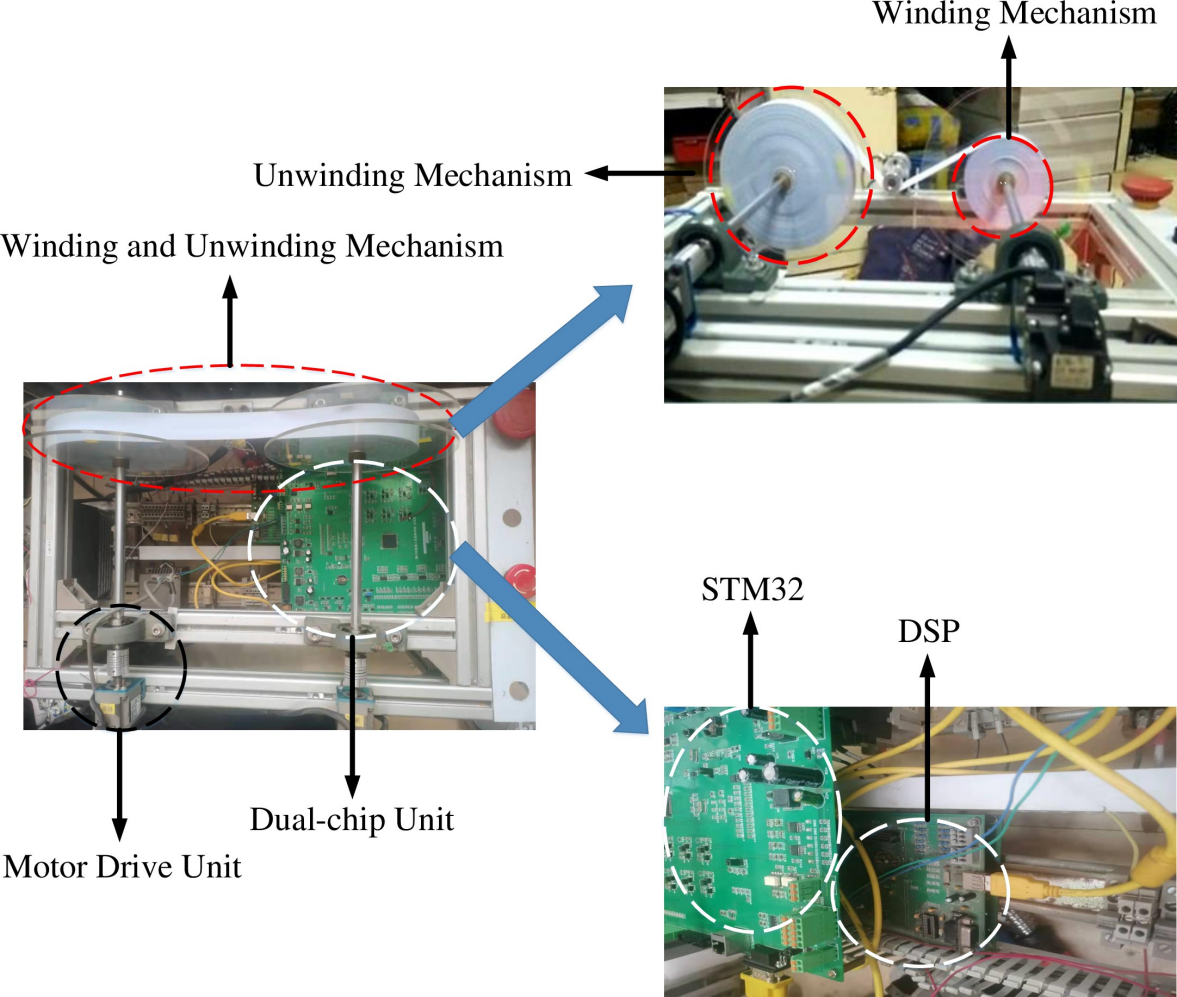
Tension control testing experiment.

After the integrated design scheme is introduced in this system, the Fuzzy PID control strategy is used to control the tension. The tension real experiment platform is shown in Fig 22. Two servo motor drive units are used to drive the conveyor belt to work, and the conveyor belt is a model for winding and unwinding. The tension is controlled by adjusting the motor speed. Adjusting the relationship between tension and winding and unwinding motor speed, so that the two achieve dynamic equilibrium. DSP collects tension information and transmits through SPI bus, tension data.

During the lithium battery electrode rolling process, the tension is preset, and the tension should be in the range of 0–200 N. When conducting the tension test, a tension setting of 100 N is used as an example, and the upper limit of tension is set to 110 N and the lower limit to 90 N.

**Fig 23:**
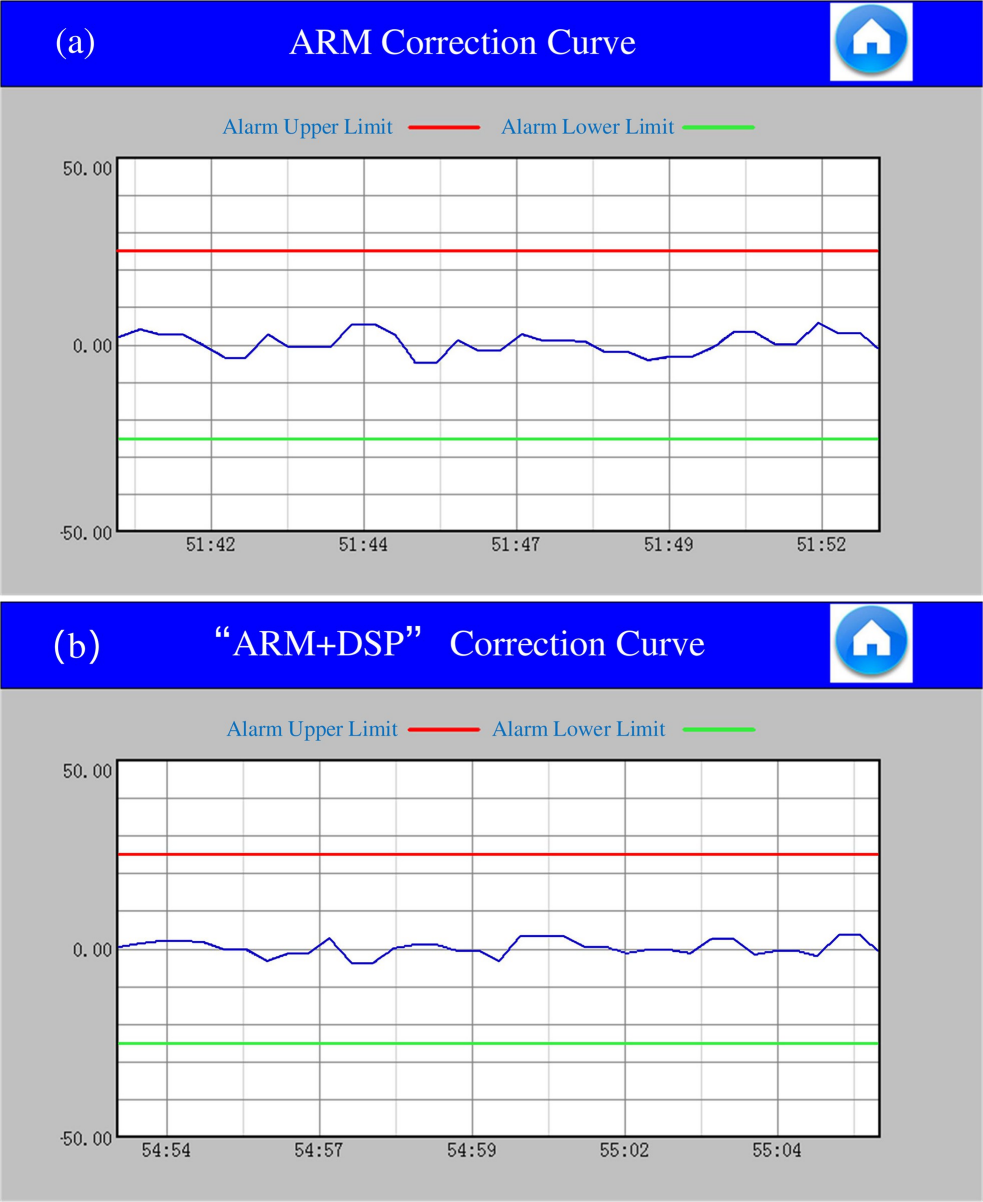
(a) ARM deflection control curve, (b) "ARM+DSP" deflection control curve.

Take the unwinding mechanism as an example, the results of the tension information are shown in Fig 23, where the tension fluctuation range for both control architectures is between 10% and stays within the set range. The tension of the integrated single-chip architecture is significantly disturbed, although it is between the upper and lower limits, which is not conducive to actual production. The "ARM+DSP" control architecture, on the other hand, has a smaller tension fluctuation range and is more stable. As can be seen from Fig 23(A), the single-chip control method has a tension sampling point closer to the set alarm boundary line, and once the disturbance increases, the tension may be too large or too small.

Ten sets of sampling points were randomly extracted from the sampling data as shown in Table 5. *T*_*u*_ is the setting of unwinding tension, $T_{u}^{'}$ is the measurement of unwinding tension in "ARM" control architecture, $T_{u}^{''}$ is the measurement of unwinding tension in "ARM+DSP" control architecture. By analyzing the deviation of tension value in the table, it can be seen that the tension error of single-chip control is within 10%, but the tension is more easily and obviously affected by external factors after system integration, while the tension error of dual chip control architecture is within 5%. Therefore, the control method of "ARM+DSP" architecture is more resistant to external disturbances and helps to improve the production quality of the pole piece.

**Table 5:** Comparison Table of unwinding tension error.

Numbers	*T*_*u*_ /N	$T_{u}^{'}$ /N	Relative Error /%	$T_{u}^{''}$ /N	Relative Error /%
1	100	93.88	6.12	102.56	2.56
2	100	95.64	4.36	101.37	1.37
3	100	103.91	3.91	98.08	1.92
4	100	94.70	5.30	103.45	3.45
5	100	101.68	1.68	101.28	1.28
6	100	107.43	7.43	102.88	2.88
7	100	99.41	0.59	99.57	0.43
8	100	104.37	4.37	101.78	1.78
9	100	102.25	2.25	100.92	0.92
10	100	96.77	3.23	101.58	1.58

### Deflection control testing

When there is no offset of the pole strip, the position of the correction sensor is corrected and the corrected position is set to the standard value of the pole strip position 0. When the pole strip position changes and swings in and out with respect to the correction sensor, the corresponding standard value is taken as positive or negative.

**Fig 24:**
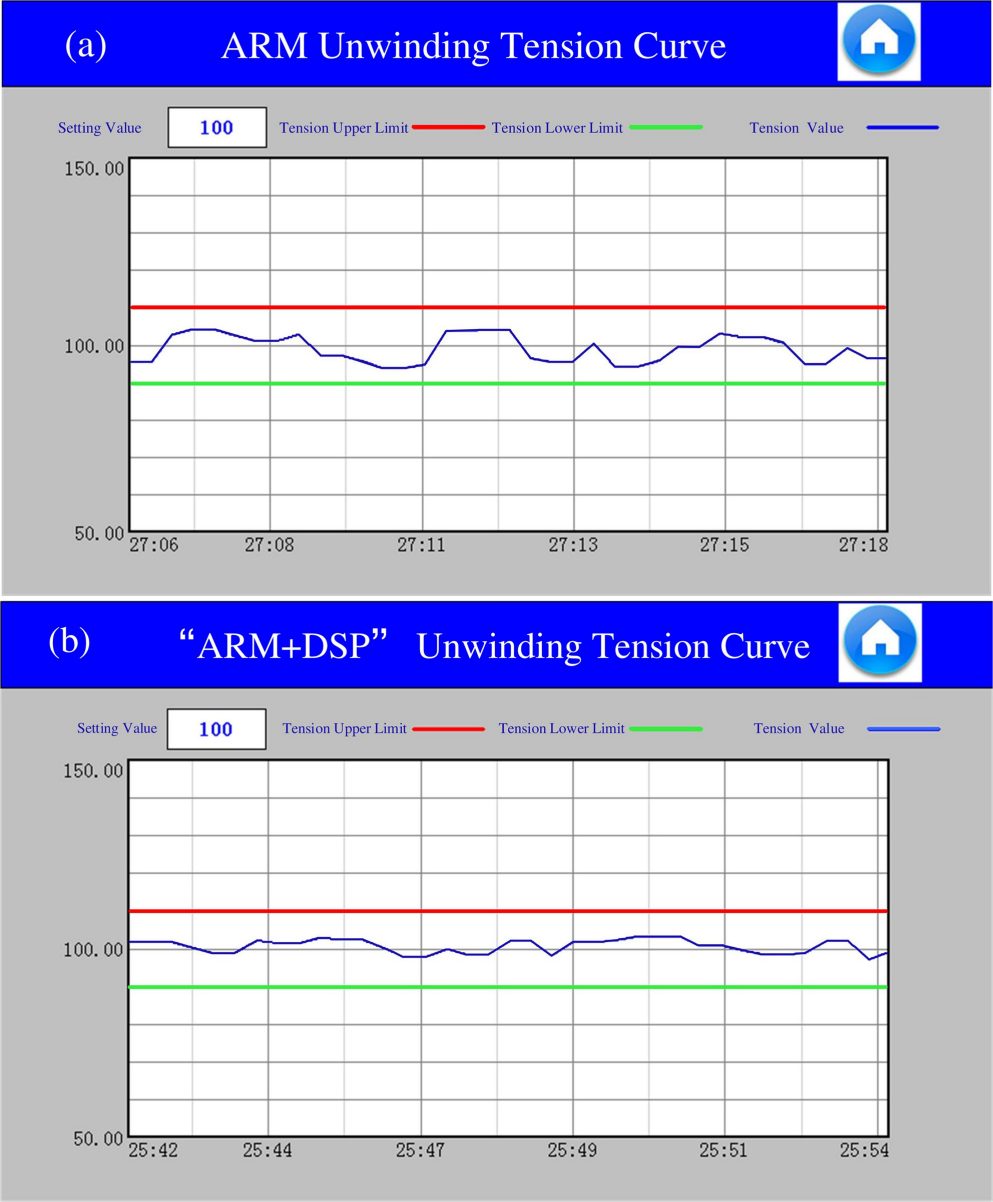
(a) ARM deflection control curve, (b) "ARM+DSP" deflection control curve.

After the equipment is running stably, the edge information of the pole strip in the deflection control is collected. Under the Fuzzy PID control method, for example, the pole strip position information of the "ARM" control architecture is shown in Fig 24(A). In the case of "ARM+DSP" control architecture, the position information of the pole strip is shown in Fig 24(B).

The collected edge information of the pole strip fluctuates up and down around the standard value of 0. When the pole strip position fluctuates under the control of STM32, the correction speed is slower and the fluctuation degree is more drastic. In contrast, under the joint control of STM32 and DSP, the adjustment speed is faster and the position information fluctuates less after the position of the pole strip deviates from the set value. In the sample data collected from the pole strip position information, the sampling points are selected at equal time intervals, and 10 sets of position sampling information are selected for each of the two methods. In Table 6, "+" and "-" indicate the direction in which the edge of the pole piece is shifted relative to the set value, *L* is the setting of pole position, *L*' is the measurement of pole position in "ARM" control architecture, *L*'' is the measurement of pole position in "ARM+DSP" control architecture.

**Table 6:** Comparison table of unwinding and deviation correction errors.

Numbers	*L* /mm	*L*' /mm	*L*'' /mm
1	50	+6.1	-3.2
2	50	+4.7	-1.7
3	50	+1.3	+2.5
4	50	-3.6	+1.3
5	50	-5.9	-1.2
6	50	-8.9	-2.6
7	50	+0.6	-0.8
8	50	+4.2	+1.9
9	50	-1.8	+2.9
10	50	+3.5	+0.6

By comparing the data in the table, the following conclusions can be drawn:

- The deviation values of the polar strip for the dual-chip control architecture are generally smaller than those for the single-chip control architecture,
- The deviation of the mid-pole strip position is within 10 ±mm in the single-chip architecture, while the deviation of the pole strip position is within 4 ±mm in the dual-chip control architecture.

Under the control mode of "ARM + DSP" architecture, the position of pole strip is more stable, the deviation correction control process is stable and reliable, and the adverse impact caused by pole strip deviation can be reduced.

## Discussion

In order to improve the operation efficiency of the existing mill control system, this paper proposes a digital integrated control system based on the dual-chip architecture of "ARM+DSP". The system separates the main drive mechanism and the winding mechanism at the physical layer, uses an isolated power supply to suppress the impact of the grid current, and incorporates a deflection unit to reduce the error in the production of pole pieces. The comparison verifies the effectiveness of Fuzzy PID theory in deflection and tension control. The results show that the tension control accuracy is higher and the correction error is lower with the dual-chip architecture.

The dual-chip architecture of "ARM+DSP" can improve the stability of the system to a certain extent. Compared to the pole mill control system proposed by the authors in [12], the dual-chip system-level architecture is able to integrate multiple functional modules. SoC (system on chip) system is a micro system, which is a special chip developed for customer needs. SoC system level chips have been used in power control [35], protection control [43] and other fields. SoC system level chips are expensive and lack of reconfigurability, so they are not suitable for nonlinear systems such as mechanical equipment. The control accuracy of the system level architecture proposed in this paper is lower than that of SoC special chip, but considering the cost and reconfigurability, the embedded system with dual chip architecture has more far-reaching research significance and is more suitable for the industrial field. The experimental test shows that the hardware platform has strong anti-interference ability, eliminates the peak, sharp frequency and other interference signals, and the signal output is stable. However, due to the experimental conditions, the following problems and areas for further improvement still exist:

- The signals of ARM and DSP interfere with each other, and the shielded line technology still does not achieve better results, Compared with SoC integrated chips, the cascade structure of "ARM+DSP" is not integrated enough and heat dissipation is not strong. In the future, the chip integration technology can be applied, combined with HPL, MailBox, OpenCL, DCE and other communication protocols to ensure stable data transmission.
- During the experimental test phase, there is a discrepancy between the tension setting value and the actual tension value in the industrial field. In the real situation, the tension will change depending on the pole piece. In the future research process, several actual working parameters can be extended to verify the tension control effect of the algorithm from several perspectives.
- The selection of deflection and tension control algorithms In addition, there are some intelligent algorithms, such as genetic self-anti-disturbance control and model predictive control, which can also be used for pole mill deflection and tension control. Therefore, based on the research in this paper, other algorithms can be investigated in the subsequent research process.

### Summary

The working environment of lithium battery pole mill is complex and the signal acquisition is difficult, and the integration and flexibility of the measurement and control system composed of single control unit such as PLC and ARM are poor, especially for the multi-motor control of complex electromechanical equipment. Therefore, this paper uses the powerful data storage and processing capability of DSP and the richness of STM32 peripherals to design a "dual-chip-three-unit" cascade control system. It is verified that the dual-chip architecture is possible for industrial field applications, and the contributions of this study are as follows:

- In this paper, the influence of pole position deviation on tension is considered. In the design of control algorithm, the influence of pole position deviation on tension is considered to improve the stability of the system
- Fuzzy PID is used to optimize the pole piece deviation correction control and tension control process, and the relevant knowledge of the influence of deviation correction control on tension control is added to the knowledge base. It is verified that fuzzy PID can effectively improve the anti-interference ability and adaptive ability of the system,
- The experimental platform of "ARM+DSP" dual-chip control was built, and the visualization interface was designed to monitor the operation status of the lithium battery pole mill. The experimental results show that the dual-chip control system operates stably, the accuracy of tension system is 5%, and the deviation of deflection system is ±4mm.

## Supporting information

S1 File (DOCX)

## References

[1] Wu X K et al. “Analysis and research on the status quo of standardization of lithium-ion battery manufacturing equipment,” New Material Industry, vol. 04, pp. 37–41. 2020.

[2] King Sarah, Naomi J Boxall. “Lithium battery recycling in Australia: defining the status and identifying opportunities for the development of a new industry,” Journal of Cleaner Production, vol. 2151, no. 4, pp. 1279–1287. 2019.

[3] Pablo B et al. “Probabilistic assessment of the impact of electric vehicles and nonlinear loads on power quality in residential networks,” International Journal of Electrical Power & Energy Systems, vol. 129, pp. 106807–106820. 2021.

[4] Qiao D et al. “Potential impact of the end-of-life batteries recycling of electric vehicles on lithium demand in China: 2010–2050,” Sci Total Environ, vol. 764, pp. 142835. 2021. doi: 10.1016/j.scitotenv.2020.142835 33097265

[5] Song D Y et al. “Design of control system for power lithium-ion battery pole piece rolling mill based on STM32,” Instrument Technology and Sensors, vol. 06, pp. 83–86. 2020.

[6] Voronin S. S., Gasiyarov V. R. and Maklakova E. A. “Modelling of the hydraulic work roll bending control system of the plate rolling mill,” presented at IEEE Conference of Russian Young Researchers in Electrical and Electronic Engineering, pp.1060–1063.2017.

[7] Wu C et al. “Analysis of the thermal contraction of wide-thick continuously cast slab and the weighted average method to design a roll gap,” Steel Research International, 2017.

[8] Sun JNA, Xiang WJ. Research progress of lithium battery electrode rolling technology [J]. China Metallurgy, 2021,31(05):12–18.

[9] Chen Y A. “Study on the progress of tension control technology of tape slitter at home and abroad,” Journal of Beijing Institute of Printing, vol. 26, no. 3, pp. 33–38, 2018.

[10] Brodd Ralph J, Carlos Helou. “Cost comparison of producing high-performance Li-ion batteries in the U.S. and in China,” Journal of Power Sources, vol. 2311, no. 6, pp. 293–300, 2011.

[11] Kornas Thomas et al. “A Multivariate KPI-Based Method for Quality Assurance in Lithium-Ion-Battery Production,” Procedia CIRP, vol. 81, pp. 75–80, 2019.

[12] Zhao H C. “Research on Embedded Control System of Lithium Battery Pole Piece Rolling Mill”. M.S. Hebei University of Technology, China, 2018.

[13] Yin F. et al. “Discrete Model Predictive Control Scheme for an Integrated Gauge-Looper Control System in a Tandem Hot Strip Mill,” IEEE Access, vol.8, pp. 73972–73985, 2020.

[14] Zhang Ze Yu, Yi Xia. “Design and Implementation of the intelligent controller for Electric Ship,” J. Phys: Conf. Ser. vol. 1639, 2020.

[15] Singh C. Perera, Kish G.J., Salmon J. “PWM Control of a Dual Inverter Drive using a Floating Capacitor Inverter”.2019 20th Workshop on Control and Modeling for Power Electronics (COMPEL), Toronto, Canada, 2019, pp.1–8.

[16] Suresh S, Rajeevan PP. “Virtual Space Vector-Based Direct Torque Control Schemes for Induction Motor Drives,” IEEE TRANSACTIONS ON INDUSTRY APPLICATIONS, vol. 56, no. 3, pp. 2719–2728, 2020.

[17] Wang T F et al. “Design of AC Speed Regulation System Based on DSP”. Shanxi Electronic Technology, vol. 05, pp. 3–5, 2020.

[18] Chen F H et al. “Design of IPMSM vector control system for electric vehicles based on DSP,” Mechatronic Engineering Technology, vol. 49, no. 11, pp. 198–200, 2020.

[19] Qi C et al. “Design of Economical AC Servo Motor Controller Based on STM32,” Industrial Instruments and Automation Devices, vol. 05, pp. 118–122, 2020.

[20] Zhang F C et al. “The Design of Controller for BLDC Based on STM32,” IOP Conf. Ser.: Earth Environ, vol. 466, 2020.

[21] Fu Q et al. “Design and test of dual-chip integrated digital silicon gyroscope interface ASIC,” Journal of Harbin Institute of Technology, vol. 49, no. 10, pp. 90–94, 2017.

[22] Rogié Brice et al. “Multi-port dynamic compact thermal models of dual-chip package using model order reduction and metaheuristic optimization,” Microelectronics Reliability, vol. 87, pp. 222–231, 2018.

[23] Liu Y et al. “Design of precision temperature control system for fiber optic gyroscope based on dual MSP430 chips,” Science and Technology Plaza, vol. 11, pp. 116–121, 2014.

[24] Chen P H, Cheng H C. “A Fully Integrated Step-Down Switched-Capacitor DC-DC Converter With Dual Output Regulation Mechanisms,” IEEE TRANSACTIONS ON CIRCUITS AND SYSTEMS II-EXPRESS BRIEFS, vol. 67, no. 9, pp. 1649–1653, 2020.

[25] Yang Y Q. “Design of multi-channel stepping motor control system based on STM32 and FPGA,” M.S. Southwest Jiaotong University, China, 2017.

[26] Yang X B et al. “Design of low power consumption mode of pure electric vehicle controller based on dual chips,” Henan Science and Technology Association: Henan Automotive Engineering Society, vol. 2, 2017.

[27] Caizzone, Stefano et al. “Multi-Chip RFID Antenna Integrating Shape-Memory Alloys for Detection of Thermal Thresholds,” IEEE Transactions on Antennas and Propagation, vol. 59, no. 7, pp. 2488–2494, 2011.

[28] Occhiuzzi, Cecilia et al. “RFID-Based Dual-Chip Epidermal Sensing Platform for Human Skin Monitoring,” IEEE Sensors Journal, vol. 21, no. 4, pp. 5359–5367, 2021.

[29] Jeon B et al. “Enhanced predictive capacity using dual-parameter chip model that simulates physiological skin irritation,” Toxicol In Vitro, vol. 68, pp. 104955, 2020. doi: 10.1016/j.tiv.2020.104955 32739441

[30] Chung W. et al, “A maximum power point tracking and voltage regulated dual-chip system for single-cell photovoltaic energy harvesting,” International Symposium on Integrated Circuits (ISIC), pp. 5–8, 2014.

[31] Han, Jiang et al. “A novel gear machining CNC design and experimental research,” The International Journal of Advanced Manufacturing Technology, vol. 88, pp. 1711–1722, 2016.

[32] Li H, Liang R. “Design of EDM wire-cutting machine control system based on embedded ARM and DSP,” Manufacturing Automation, vol. 41, no. 11, pp. 84–87, 2019.

[33] Song W Q. “Design of can product sorting system based on dynamic weighing technology,” M.S. Harbin Institute of Technology, China, 2021.

[34] Tong W M, Chen P Y. “Design of power quality detection device based on DSP+ARM dual-core system,” Electrical Measurement and Instrumentation, vol. 56, no. 18, pp. 99–106, 2019.

[35] Dave Rasesh et al. “Development of control system for multi-converter High voltage Power supply using programmable SoC,” Journal of Physics: Conference Series, vol. 823, pp. 12035–12043, 2017.

[36] You B, “Research on strip runout and deflection correction in acid rolling units,” Yanshan University, China, 2017.

[37] Yang G F, “Fuzzy PID-based adaptive edge deflection correction system for flexible packaging strips,” Packaging and Food Machinery, vol. 37, pp. 04, pp. 43–46, 2019.

[38] Zhang D Y et al, “Self-anti-disturbance control of strip guiding system,” New Technology New Process, vol. 01, pp. 42–47, 2018.

[39] Jiang C, Wang H, Hou L et al. “Sliding Mode Compensation Control for Diaphragm Tension in Unwinding Process of Lithium Battery Diaphragm Slitting Machine,” IEEE Access, 2020.

[40] Ashour H, Abd-Elraouf M, El-Shenawy A. “Practical validation of PLC-based sensor-less winder tension control,” 2019 IEEE International Conference on Environment and Electrical Engineering and 2019 IEEE Industrial and Commercial Power Systems Europe (EEEIC / I&CPS Europe), 2019.

[41] Shokhin O.V. Permyakova, “The Study of Continuous Rolling Mill Inter-stand Tension Inferential Control Systems,” Procedia Engineering, vol. 29, 2015.

[42] Wang Y K. “Development and implementation of an all-electric injection molding machine control system based on ARM and DSP,” South China University of Technology, China, 2019.

[43] Zou, X F et al. “Research on Substation Protection Measurement and Control Integrated Configuration Device Based on SoC Chip”, IOP Conference Series: Materials Science and Engineering, vol. 768, no. 6, pp. 062054, 2020.

[44] Wang L G. “Analysis and simulation of strip deviation electro-hydraulic position servo control system,” International Conference on Electronics, IEEE, 2011.

[45] Sun J N et al. “Research Progress of Rolling Technology of Lithium Battery Pole Pieces,” China Metallurgical, vol. 31, no. 05, pp. 12–18, 2021.

[46] Mourtzis, Dimitris, “Simulation in the design and operation of manufacturing systems: state of the art and new trends,” International Journal of Production Research, vol. 58, no. 7, pp. 1927–1949, 2020.

[47] Heidari A, Forouzan M R, Niroomand M R. “Development and evaluation of friction models for chatter simulation in cold strip rolling,” The International Journal of Advanced Manufacturing Technology, vol. 96, pp. 2055–2075, 2018.

[48] Ma S, Tian L, “Stiffness analysis and structure optimization of rolling mill for lithium-ion battery electrode manufacturing,” Mechanical Engineering, vol. 26, no. 6, pp. 803–808, 2015.

[49] Lu J S, Chen H R, Cheng M Y et al. “Tension control improvement in automatic stator in-slot winding machines using iterative learning control,” International Conference on Information Science. IEEE, 2014.

