# Supplementary material for: Optimized design of battery pole control system based on dual-chip architecture
Source: PLoS One. 2022 May 11;17(5):e0264285. doi: 10.1371/journal.pone.0264285 (PMC9094561; doi:10.1371/journal.pone.0264285)
Supplement: S1 File — (DOCX) [file pone.0264285.s001.docx]

The datasets we provide include the minimal dataset underlying the results of our study.

All experimental data are shown in Table 5 and Table 6，Table 5 shows the data of tension detection and control testing, and Table 6 shows the data of deflection control testing.$I_{in}$ is the inlet thickness of the pole piece, $I_{out}$ is the outlet thickness of the pole piece, $W$ is the inlet width of the pole piece, $V$ is the speed of rolling, $F$ is the rolling force, $T_{w}$ is the setting of winding tension, $T_{w}^{'}$ is the measurement of winding tension in "ARM" control architecture, $T_{u}$ is the setting of unwinding tension, $T_{u}^{'}$ is the measurement of unwinding tension in "ARM" control architecture, $S$ is the roll gap, $L$ is the setting of pole position, $L^{'}$ is the measurement of pole position in "ARM" control architecture. $T_{w}^{''}$ is the measurement of winding tension in "ARM+DSP" control architecture. $T_{u}^{''}$ is the measurement of unwinding tension in "ARM+DSP" control architecture. $L^{''}$ is the measurement of pole position in "ARM+DSP" control architecture.

A total of 100 sets of data are actually measured in the Table 5 and Table 6. We randomly selects 10 sets of tension detection and control testing data and 10 sets of deflection control testing data, and then analyzes the selected data in Table 5 and Table 6.

|  | | Table 5 Experimental data of tension detection and control testing | | | | | | | | |  | |  |
| --- | --- | --- | --- | --- | --- | --- | --- | --- | --- | --- | --- | --- | --- |
| $I_{in}$(mm) | $I_{out}$(mm) | | $W$  (mm) | $V$ (mm/s) | $F$  (t) | $T_{w}$  (N) | $T_{w}^{'}$  (N) | $T_{w}^{''}$  (N) | $T_{u}$  (N) | $T_{u}^{'}$  (N) | | $T_{u}^{''}$  (N) | |
| 0.1812 | 0.1197 | | 500.75 | 698 | 61.7 | 100 | 93.88 | 101.33 | 100 | 99.98 | | 101.62 | |
| 0.1791 | 0.1193 | | 499.86 | 700 | 61.9 | 100 | 95.64 | 99.84 | 100 | 104.59 | | 100.64 | |
| 0.1791 | 0.1200 | | 500.27 | 698 | 59.3 | 100 | 103.91 | 98.60 | 100 | 98.49 | | 100.45 | |
| 0.1814 | 0.1185 | | 500.14 | 700 | 60.5 | 100 | 94.70 | 102.91 | 100 | 97.85 | | 101.25 | |
| 0.1829 | 0.1199 | | 500.25 | 702 | 56.4 | 100 | 101.68 | 98.11 | 100 | 96.29 | | 99.49 | |
| 0.1774 | 0.1197 | | 500.70 | 703 | 62.4 | 100 | 107.43 | 98.89 | 100 | 104.59 | | 98.93 | |
| 0.1820 | 0.1198 | | 500.44 | 699 | 63.9 | 100 | 99.41 | 100.30 | 100 | 100.47 | | 98.15 | |
| 0.1811 | 0.1194 | | 499.99 | 700 | 59.1 | 100 | 104.37 | 100.94 | 100 | 96.96 | | 101.10 | |
| 0.1777 | 0.1195 | | 499.62 | 701 | 63.4 | 100 | 102.25 | 101.65 | 100 | 102.57 | | 98.41 | |
| 0.1828 | 0.1195 | | 500.80 | 699 | 64.8 | 100 | 96.77 | 100.12 | 100 | 100.68 | | 102.05 | |
| 0.1806 | 0.1200 | | 500.10 | 700 | 64.3 | 100 | 93.88 | 98.64 | 100 | 98.40 | | 101.70 | |
| 0.1771 | 0.1195 | | 500.85 | 700 | 62.1 | 100 | 95.64 | 102.90 | 100 | 96.38 | | 101.43 | |
| 0.1797 | 0.1209 | | 499.91 | 699 | 63.5 | 100 | 104.57 | 99.13 | 100 | 97.51 | | 100.27 | |
| 0.1823 | 0.1210 | | 499.91 | 698 | 60.9 | 100 | 102.92 | 99.71 | 100 | 102.53 | | 97.71 | |
| 0.1813 | 0.1187 | | 499.28 | 702 | 62.7 | 100 | 101.55 | 99.56 | 100 | 99.69 | | 101.99 | |
| 0.1794 | 0.1182 | | 500.38 | 701 | 64.0 | 100 | 104.50 | 102.99 | 100 | 100.87 | | 99.82 | |
| 0.1771 | 0.1205 | | 500.83 | 703 | 56.8 | 100 | 102.09 | 98.78 | 100 | 96.49 | | 98.45 | |
| 0.1828 | 0.1195 | | 500.80 | 699 | 64.8 | 100 | 104.64 | 99.92 | 100 | 101.16 | | 99.55 | |
| 0.1823 | 0.1210 | | 499.91 | 698 | 60.9 | 100 | 104.59 | 100.92 | 100 | 98.80 | | 100.64 | |
| 0.1774 | 0.1197 | | 500.70 | 703 | 62.4 | 100 | 96.71 | 98.80 | 100 | 95.11 | | 98.53 | |
| 0.1811 | 0.1194 | | 499.99 | 700 | 59.1 | 100 | 95.34 | 98.85 | 100 | 97.23 | | 97.21 | |
| 0.1777 | 0.1195 | | 499.62 | 701 | 63.4 | 100 | 102.54 | 102.27 | 100 | 97.57 | | 102.50 | |
| 0.1828 | 0.1195 | | 500.80 | 699 | 64.8 | 100 | 96.57 | 100.91 | 100 | 99.73 | | 100.86 | |
| 0.1806 | 0.1200 | | 500.10 | 700 | 64.3 | 100 | 104.57 | 98.53 | 100 | 100.67 | | 99.70 | |
| 0.1784 | 0.1213 | | 499.53 | 702 | 58.6 | 100 | 102.92 | 98.89 | 100 | 98.37 | | 100.68 | |
| 0.1801 | 0.1182 | | 500.28 | 701 | 56.8 | 100 | 101.55 | 98.16 | 100 | 102.51 | | 98.05 | |
| 0.1814 | 0.1190 | | 500.69 | 702 | 58.3 | 100 | 104.50 | 101.22 | 100 | 103.40 | | 98.61 | |
| 0.1771 | 0.1189 | | 499.01 | 701 | 58.3 | 100 | 102.09 | 99.25 | 100 | 98.51 | | 100.88 | |
| 0.1795 | 0.1203 | | 499.52 | 701 | 56.9 | 100 | 104.64 | 102.53 | 100 | 95.75 | | 99.75 | |
| 0.1786 | 0.1215 | | 500.55 | 702 | 56.0 | 100 | 101.55 | 100.11 | 100 | 96.62 | | 100.49 | |
| 0.1775 | 0.1205 | | 499.50 | 700 | 61.5 | 100 | 102.06 | 100.80 | 100 | 97.55 | | 101.33 | |
| 0.1796 | 0.1194 | | 499.83 | 702 | 62.2 | 100 | 99.38 | 99.88 | 100 | 97.54 | | 101.59 | |
| 0.1815 | 0.1206 | | 500.73 | 697 | 62.7 | 100 | 97.76 | 99.45 | 100 | 103.30 | | 101.07 | |
| 0.1809 | 0.1218 | | 500.86 | 701 | 62.1 | 100 | 104.70 | 102.39 | 100 | 95.53 | | 100.97 | |
| 0.1784 | 0.1213 | | 499.53 | 702 | 58.6 | 100 | 95.35 | 98.47 | 100 | 102.94 | | 100.24 | |
| 0.1801 | 0.1182 | | 500.28 | 701 | 56.8 | 100 | 95.31 | 102.40 | 100 | 100.05 | | 99.84 | |
| 0.1819 | 0.1207 | | 499.92 | 699 | 58.6 | 100 | 98.81 | 98.95 | 100 | 103.14 | | 98.13 | |
| 0.1817 | 0.1182 | | 499.12 | 698 | 58.8 | 100 | 101.79 | 101.08 | 100 | 100.85 | | 100.81 | |
| 0.1771 | 0.1204 | | 499.37 | 697 | 63.5 | 100 | 104.57 | 102.08 | 100 | 100.30 | | 101.62 | |
| 0.1801 | 0.1200 | | 499.04 | 697 | 62.4 | 100 | 103.49 | 100.99 | 100 | 98.11 | | 102.21 | |
| 0.1792 | 0.1215 | | 499.25 | 697 | 57.5 | 100 | 97.76 | 101.34 | 100 | 101.99 | | 97.91 | |
| 0.1775 | 0.1202 | | 499.24 | 700 | 57.3 | 100 | 102.65 | 100.14 | 100 | 97.43 | | 98.72 | |
| 0.1826 | 0.1196 | | 500.92 | 698 | 64.2 | 100 | 101.55 | 99.32 | 100 | 100.49 | | 102.67 | |
| 0.1784 | 0.1188 | | 499.22 | 699 | 55.1 | 100 | 99.85 | 99.30 | 100 | 102.79 | | 99.10 | |
| 0.1810 | 0.1197 | | 500.37 | 698 | 55.4 | 100 | 104.33 | 100.35 | 100 | 100.28 | | 98.58 | |
| 0.1823 | 0.1201 | | 499.87 | 697 | 63.6 | 100 | 95.46 | 98.95 | 100 | 103.90 | | 99.04 | |
| 0.1799 | 0.1215 | | 499.50 | 699 | 56.4 | 100 | 102.95 | 100.41 | 100 | 104.29 | | 97.54 | |
| 0.1786 | 0.1200 | | 499.05 | 699 | 55.2 | 100 | 96.62 | 102.12 | 100 | 104.17 | | 98.25 | |
| 0.1806 | 0.1195 | | 499.89 | 701 | 56.4 | 100 | 103.07 | 100.97 | 100 | 104.34 | | 100.97 | |
| 0.1807 | 0.1198 | | 500.09 | 700 | 57.9 | 100 | 101.78 | 101.47 | 100 | 96.65 | | 98.90 | |
| 0.1814 | 0.1190 | | 500.69 | 702 | 58.3 | 100 | 95.97 | 101.33 | 100 | 99.98 | | 101.62 | |
| 0.1771 | 0.1189 | | 499.01 | 701 | 58.3 | 100 | 96.86 | 99.84 | 100 | 104.59 | | 100.64 | |
| 0.1795 | 0.1203 | | 499.52 | 701 | 56.9 | 100 | 96.19 | 98.60 | 100 | 98.49 | | 100.45 | |
| 0.1786 | 0.1215 | | 500.55 | 702 | 56.0 | 100 | 101.55 | 102.91 | 100 | 97.85 | | 101.25 | |
| 0.1775 | 0.1205 | | 499.50 | 700 | 61.5 | 100 | 102.06 | 98.11 | 100 | 96.29 | | 99.49 | |
| 0.1796 | 0.1194 | | 499.83 | 702 | 62.2 | 100 | 99.38 | 98.89 | 100 | 104.59 | | 98.93 | |

|  | | Table 6 Experimental data of deflection control testing | | | | | | | |  |
| --- | --- | --- | --- | --- | --- | --- | --- | --- | --- | --- |
| $I_{in}$(mm) | $I_{out}$(mm) | | $W$  (mm) | $V$ (mm/s) | $F$  (t) | $S$ (mm) | $L$  (mm) | $L^{'}$  (mm) | $L^{''}$  (mm) | |
| 0.1812 | 0.1197 | | 500.75 | 698 | 61.7 | 0.038 | 50 | 49.2 | 52.5 | |
| 0.1791 | 0.1193 | | 499.86 | 700 | 61.9 | 0.042 | 50 | 52.8 | 46.5 | |
| 0.1791 | 0.1200 | | 500.27 | 698 | 59.3 | 0.045 | 50 | 53.3 | 52.8 | |
| 0.1814 | 0.1185 | | 500.14 | 700 | 60.5 | 0.038 | 50 | 42.4 | 45.9 | |
| 0.1829 | 0.1199 | | 500.25 | 702 | 56.4 | 0.035 | 50 | 41.3 | 50.8 | |
| 0.1774 | 0.1197 | | 500.70 | 703 | 62.4 | 0.039 | 50 | 43.4 | 52.0 | |
| 0.1820 | 0.1198 | | 500.44 | 699 | 63.9 | 0.036 | 50 | 48.7 | 46.8 | |
| 0.1811 | 0.1194 | | 499.99 | 700 | 59.1 | 0.043 | 50 | 41.6 | 48.5 | |
| 0.1777 | 0.1195 | | 499.62 | 701 | 63.4 | 0.038 | 50 | 53.3 | 47.3 | |
| 0.1828 | 0.1195 | | 500.80 | 699 | 64.8 | 0.044 | 50 | 43.9 | 48.9 | |
| 0.1806 | 0.1200 | | 500.10 | 700 | 64.3 | 0.042 | 50 | 49.1 | 49.4 | |
| 0.1771 | 0.1195 | | 500.85 | 700 | 62.1 | 0.040 | 50 | 54.4 | 46.3 | |
| 0.1797 | 0.1209 | | 499.91 | 699 | 63.5 | 0.041 | 50 | 45.9 | 45.8 | |
| 0.1823 | 0.1210 | | 499.91 | 698 | 60.9 | 0.036 | 50 | 48.9 | 47.5 | |
| 0.1813 | 0.1187 | | 499.28 | 702 | 62.7 | 0.041 | 50 | 41.4 | 49.6 | |
| 0.1794 | 0.1182 | | 500.38 | 701 | 64.0 | 0.039 | 50 | 50.3 | 49.9 | |
| 0.1771 | 0.1205 | | 500.83 | 703 | 56.8 | 0.043 | 50 | 41.7 | 46.8 | |
| 0.1828 | 0.1195 | | 500.80 | 699 | 64.8 | 0.044 | 50 | 43.9 | 47.0 | |
| 0.1823 | 0.1210 | | 499.91 | 698 | 60.9 | 0.036 | 50 | 48.4 | 48.3 | |
| 0.1774 | 0.1197 | | 500.70 | 703 | 62.4 | 0.039 | 50 | 42.5 | 46.8 | |
| 0.1811 | 0.1194 | | 499.99 | 700 | 59.1 | 0.043 | 50 | 51.2 | 49.6 | |
| 0.1777 | 0.1195 | | 499.62 | 701 | 63.4 | 0.038 | 50 | 46.4 | 48.4 | |
| 0.1828 | 0.1195 | | 500.80 | 699 | 64.8 | 0.044 | 50 | 52.0 | 48.2 | |
| 0.1806 | 0.1200 | | 500.10 | 700 | 64.3 | 0.042 | 50 | 42.7 | 49.0 | |
| 0.1784 | 0.1213 | | 499.53 | 702 | 58.6 | 0.045 | 50 | 43.59 | 48.6 | |
| 0.1801 | 0.1182 | | 500.28 | 701 | 56.8 | 0.041 | 50 | 46.5 | 46.6 | |
| 0.1814 | 0.1190 | | 500.69 | 702 | 58.3 | 0.038 | 50 | 41.9 | 47.4 | |
| 0.1771 | 0.1189 | | 499.01 | 701 | 58.3 | 0.038 | 50 | 46.7 | 49.7 | |
| 0.1795 | 0.1203 | | 499.52 | 701 | 56.9 | 0.043 | 50 | 52.9 | 45.6 | |
| 0.1786 | 0.1215 | | 500.55 | 702 | 56.0 | 0.037 | 50 | 46.5 | 52.7 | |
| 0.1775 | 0.1205 | | 499.50 | 700 | 61.5 | 0.038 | 50 | 41.5 | 47.4 | |
| 0.1796 | 0.1194 | | 499.83 | 702 | 62.2 | 0.040 | 50 | 54.2 | 52.3 | |
| 0.1815 | 0.1206 | | 500.73 | 697 | 62.7 | 0.041 | 50 | 53.5 | 47.0 | |
| 0.1809 | 0.1218 | | 500.86 | 701 | 62.1 | 0.041 | 50 | 49.3 | 47.0 | |
| 0.1784 | 0.1213 | | 499.53 | 702 | 58.6 | 0.045 | 50 | 40.4 | 49.3 | |
| 0.1801 | 0.1182 | | 500.28 | 701 | 56.8 | 0.041 | 50 | 43.3 | 48.7 | |
| 0.1819 | 0.1207 | | 499.92 | 699 | 58.6 | 0.040 | 50 | 40.6 | 48.4 | |
| 0.1817 | 0.1182 | | 499.12 | 698 | 58.8 | 0.036 | 50 | 42.2 | 49.8 | |
| 0.1771 | 0.1204 | | 499.37 | 697 | 63.5 | 0.042 | 50 | 45.6 | 51.4 | |
| 0.1801 | 0.1200 | | 499.04 | 697 | 62.4 | 0.042 | 50 | 53.4 | 49.1 | |
| 0.1792 | 0.1215 | | 499.25 | 697 | 57.5 | 0.036 | 50 | 53.7 | 46.8 | |
| 0.1775 | 0.1202 | | 499.24 | 700 | 57.3 | 0.043 | 50 | 51.6 | 46.4 | |
| 0.1826 | 0.1196 | | 500.92 | 698 | 64.2 | 0.042 | 50 | 43.9 | 50.6 | |
| 0.1784 | 0.1188 | | 499.22 | 699 | 55.1 | 0.039 | 50 | 47.6 | 45.2 | |
| 0.1810 | 0.1197 | | 500.37 | 698 | 55.4 | 0.037 | 50 | 54.1 | 46.8 | |
| 0.1823 | 0.1201 | | 499.87 | 697 | 63.6 | 0.038 | 50 | 42.2 | 51.7 | |
| 0.1799 | 0.1215 | | 499.50 | 699 | 56.4 | 0.039 | 50 | 52.2 | 52.2 | |
| 0.1786 | 0.1200 | | 499.05 | 699 | 55.2 | 0.040 | 50 | 42.1 | 46.7 | |
| 0.1806 | 0.1195 | | 499.89 | 701 | 56.4 | 0.040 | 50 | 46.0 | 52.4 | |
| 0.1807 | 0.1198 | | 500.09 | 700 | 57.9 | 0.044 | 50 | 47.3 | 48.9 | |
| 0.1814 | 0.1190 | | 500.69 | 702 | 58.3 | 0.038 | 50 | 49.0 | 52.5 | |
| 0.1771 | 0.1189 | | 499.01 | 701 | 58.3 | 0.038 | 50 | 52.3 | 46.5 | |
| 0.1795 | 0.1203 | | 499.52 | 701 | 56.9 | 0.043 | 50 | 53.0 | 52.8 | |
| 0.1786 | 0.1215 | | 500.55 | 702 | 56.0 | 0.037 | 50 | 42.0 | 45.9 | |
| 0.1775 | 0.1205 | | 499.50 | 700 | 61.5 | 0.038 | 50 | 41.1 | 50.8 | |
| 0.1796 | 0.1194 | | 499.83 | 702 | 62.2 | 0.040 | 50 | 43.9 | 52.0 | |
